# CDKN1A/p21 Influences the Survival and Expansion of Breast Cancer Stem Cells after Oxidative Damage

**DOI:** 10.32604/or.2026.074965

**Published:** 2026-04-22

**Authors:** Evangelos Manousakis, Cristina Moreta-Moraleda, Clàudia Martinez Miralles, Anna Tomàs Pujolà, Houda Baccara, Laia Liñán Franquet, Montserrat Montañés Albó, Roberto Ferrari, Roni H. G. Wright

**Affiliations:** 1 Department of Biomedical Sciences, Faculty of Medicine and Health Sciences, Universitat Internacional de Catalunya (UIC), Barcelona, Spain; 2 Department of Chemistry, Life Sciences and Environmental Sustainability, University of Parma, Parma, Italy

**Keywords:** Breast cancer stem cells (BCSCs), CDKN1A/p21, gene expression, chromatin, oxidative damage, spheroid, therapy resistance

## Abstract

**Objective:**

Breast cancer remains one of the most prevalent malignancies among women worldwide, and despite advances in therapy and treatment options, tumour relapse and metastasis remain major clinical challenges, largely driven by the breast cancer stem cells (BCSCs) niche that resists conventional treatments and regenerates tumours. In breast cancer, where approximately 30% of patients who initially respond to treatment ultimately relapse and die of metastatic disease, targeting BCSCs is critical for improving patient outcomes. Cyclin-dependent kinase inhibitor 1A/p21 (CDKN1A/p21) is a multifunctional protein that is known primarily for its role in regulating the cell cycle in response to DNA damage. However, in this study, we aimed to explore the role of CDKN1A/p21 in the survival and expansion of BCSCs.

**Methods:**

We used three-dimensional *in vitro* models to assess the influence of CDKN1A/p21 expression on the survival of BCSCs both under basal conditions and after oxidative damage. Spatial transcriptomics analysis and chromatin immunoprecipitation-quantitative PCR (qPCR) were used to investigate the role of CDKN1A/p21 in the regulation and control of BCSC gene expression signatures.

**Results:**

We demonstrated that alterations in CDKN1A/p21 expression affect the ability of breast cancer cells to grow and survive after oxidative damage. Mechanistically, we found that CDKN1A/p21 directly binds to the promoter and regulates the expression of CD44, SPP1, and TMSB10, a combination gene signature that is associated with a greater probability of recurrence and metastasis in breast cancer patients.

**Conclusions:**

We propose that changes in gene regulation mediated by CDKN1A/p21 possibly contribute to cancer stem cell survival after oxidative damage, thus making CDKN1A/p21 a promising target for future drug discovery projects aimed at addressing the issue of therapeutic resistance and breast cancer metastasis.

## 1 Introduction

Breast cancer is the most prevalent cancer in women worldwide, with more than 2 million cases every year [1]. Despite advancements in treatment (radiotherapy, immunotherapy, chemotherapy, and surgery), which result in approximately a 90% 5-year survival rate for cancer detected at the earliest stages (TNM I and II), this survival rate drops dramatically to 30% for breast cancers detected at later stages (TNM IV) [2,3]. Even with an early diagnosis, 20%–30% of patients eventually die from metastatic disease. Compared with primary tumours, metastatic tumours are more aggressive and often resistant to chemotherapy and other standard care treatment options [4,5]. Cancer stem cells (CSCs) are a small population of cells residing initially within the primary tumour. CSCs are self-renewing (pluripotent) and are considered initiators of tumourigenesis, metastasis, and recurrence. CSCs are also increasingly resistant to classical therapeutic strategies [6]. Therefore, the identification of key players in cancer stem cell resistance is necessary to develop novel targeted therapies.

CDKN1A/p21 is known for its role in the cell cycle, where it induces cell cycle arrest by inhibiting the activity of cyclin-dependent kinases (CDKs) [7,8]. However, the role of CDKN1A/p21 is not limited to the role in cell cycle control, as it has been shown to be involved in a multitude of cellular processes, including apoptosis, epithelial–mesenchymal transition (EMT), and the DNA damage response [9,10], important pathways during metastatic growth. Interestingly, the role of CDKN1A/p21 in these processes can be either oncogenic or tumour-suppressing, depending on the specific process, pathway, cell type, interacting partners, and the cellular localization of the protein [11]. The dynamic fluctuations of the CDKN1A/p21 protein during the cell cycle are well established [12], and there is evidence that the increase after DNA damage mediates cell fate decisions, i.e., if the cell remains in a proliferative state or enters into senescence [13,14]. Multiple studies have highlighted the role of CDKN1A/p21 in regulating stem cell survival and expansion [15–17], and it has also been proposed to play a potential role in drug resistance [18–20]. Interestingly, CDKN1A/p21 knockdown has been shown to increase proliferation, but decrease spheroid and colony formation in breast cancer cell lines, suggesting a context-dependent role in breast cancer biology [21].

Moreover, at the level of chromatin and gene expression, CDKN1A/p21 can directly affect gene regulation either by forming multiprotein complexes with other transcription factors (TFs) [22,23] or by acting as a transcription factor itself [24,25]. Cancer stem cell (CSC) phenotypic properties are dependent on the underlying transcriptional program, TF binding, and a plethora of tightly controlled epigenetic changes [26–29]. In addition, recent findings indicate that CSC plasticity, chromatin organisation, and architecture are alternatively regulated and controlled in cancer, and perturbation of the key proteins correlates with patient response and prognosis [30]. Overall, the emerging role of CDKN1A/p21 in gene and chromatin regulation makes it an interesting candidate for mediating the transcriptional modifications necessary for CSC survival after DNA damage.

Among the key factors implicated in CSC survival is the cell surface adhesion molecule CD44. CD44 expression has been linked with increased resistance to chemotherapy [31] and has been used as a biomarker to identify cancer stem cells, especially in breast cancer [32,33]. Additionally, high *CD44* mRNA expression is correlated with poorer patient outcomes, with increased progression to metastatic disease and poorer overall survival [34–38]. For this reason, the aim of this study was to investigate the potential mechanistic relationship between CD44 and CDKN1A in BCSCs, which could provide insights into the factors driving breast cancer stem cells (BCSCs) survival after treatment.

## 2 Methods

### 2.1 Cell Culture

All cell lines used in the study; Human Breast Adenocarcinoma; MCF7, Human Breast Ductal Carcinoma; BT474, and Human Breast triple negative Breast Adenocarcinoma; MDA-231 were purchased and authenticated by STR profiling by the American Type Culture Collection (ATCC), VA, USA catalogue numbers #HTB-22, #HTB-20, and #HTB-26, respectively. Cells were cultured in high-glucose Dulbecco’s Modified Eagle Medium DMEM, (Thermo Fisher Scientific Inc., Waltham, MA, USA #D6546) supplemented with 10% fetal bovine serum (FBS) (Thermo Fisher Scientific Inc., #17479633), 1% GlutaMAX (Thermo Fisher Scientific Inc., #X0551-100) and 1% P/S (Sigma Aldrich, Merck Group, Darmstadt, Germany #P0781) under adherent conditions or in DMEM-F12 (Thermo Fisher Scientific Inc., #11320-074) supplemented with 10% FBS, 1% P/S, 20 ng/mL EGF (StemCell technologies, Vancouver, BC, Canada #78006.1) and 2% B27 (Thermo Fisher Scientific Inc., #17504044) under low-adherence conditions. The cells were maintained at 37°C and 5% CO_2_ and tested regularly for mycoplasma. UC2288 (UC) (Sigma Aldrich, Merck Group, #S328130001) was used at 0.5, 1, 1.5, 2, 5 and 10 μM. H_2_O_2_ (Sigma Aldrich, Merck Group, #Z0898497342) was used for 6 h, and the cells were washed once with PBS before the media was replaced.

To assess spheroid growth, the cells were plated in 96 V-bottom low attachment plates (Thermo Fisher Scientific Inc. #174929) at a concentration of 1000 cells/well. Eight days later, images were captured. For spheroid initiation, cells were plated in flat-bottom low-attachment plates (Corning Incorporated, Corning, NY, USA #3474) at a concentration of 1000 cells/well. Images were captured on day 14. To measure spheroid growth after DNA damage, cells were plated in 96 V-bottom low attachment plates (Thermo Fisher Scientific Inc., #174929) 4 h after transfection at a concentration of 1000 cells/well. The next day, they were treated with H_2_O_2_, and 24 h later, they were trypsinized until a single-cell mixture was obtained and plated in a new 96 V-bottom low-attachment plate. Pictures were taken 6 h after treatment and on day 12. All the images were captured via the Invitrogen^™^ EVOS^™^ M5000 Imaging System (Thermo Fisher Scientific, #AMF5000). The spheroid number and size were quantified with ImageJ (Version 1.53t, National Institutes of Health [NIH], Bethesda, MD, USA), and the spheroid size is presented as a normalized value.

The transfection of the siRNA against CDKN1A/p21 (Sigma Aldrich Merck Group, SASI_Hs01_00025255) and the p21-FLAG constructs was performed via the Lipofectamine^™^ 2000 Transfection Reagent (Invitrogen, #11668-019) and Opti-MEM^™^ I Reduced Serum Medium without phenol red (Thermo Fisher Scientific, #11058021) according to the manufacturer’s instructions. Cells were transfected at 70%–80% confluency for a duration of 4 h. The siRNA against CDKN1A/p21 was added at a final concentration of 100 nM. A non-targeting scrambled siRNA was used as a negative control. For CDKNA/p21 overexpression, the p21-FLAG WT (Addgene, MA, USA #16240), p21-FLAG T145A (Addgene, MA, USA #16241), and p21-FLAG T145D (Addgene, MA, USA #16242) plasmids all encode CDKN1A/p21 with a C-terminal DYKDDDDK (p21-FLAG) tag [39]. For overexpression experiments, 0.3 μg of p21-FLAG plasmid DNA per well in a 6-well plate was used, corresponding to a final concentration of 0.15 μg/mL.

### 2.2 Protein Expression Quantification

Total protein extracts were extracted from 4–6 × 10^6^ MCF7 and BT474 cells via NP40 lysis buffer (0.5% NP 40, 150 mM NaCl, 50 mM Tris-HCl, dH_2_O) at 60%–80% confluency. Lysates were centrifuged at 14,000 rpm for 10 min at 4°C. The protein concentration was quantified via the Pierce^™^ BCA Protein Assay Kit (Thermo Fisher Scientific Inc., #23225).

For western blotting and protein detection, 20–30 μg of protein were loaded per gel using sodium dodecyl sulfate-polyacrylamide gel electrophoresis (SDS-PAGE) was performed on 4%–15% gels (NZYtech, Lisbon, Portugal #MB46601), and the membrane was blocked with 5% milk in Tris-buffered saline with 1% Tween 20 (TBST) for 1 h at room temperature (RT). Primary antibodies diluted in 5% milk in TBST were incubated overnight at 4°C (1:1000, CDKN1A/p21, Santa Cruz Biotechnology, SCBT, Dallas, TX, USA #sc6246; 1:1000, CDKN1A/p21, Abcam, Cambridge, UK #ab109520; 1:2000, GAPDH, Santa Cruz Biotechnology, SCBT, Dallas, TX, USA, #sc 365062, and 1:5000 for hFAB^™^ Rhodamine Anti-GAPDH BioRad, Barcelona, Spain #12004168). Secondaries (1:3000 for StarBright Blue 700 goat anti-mouse IgG BioRad #12004158, 1:10000 for IgG rabbit polyclonal BioRad, Barcelona, Spain #515035003) were incubated for 1 h at room temperature. Images were taken via the ChemiDoc MP Imaging System (Bio-Rad, #12003154) and analysed via ImageJ version 1.53t (NIH). For UC2288-treated samples, Ponceau S (Sigma Aldrich, Merck Group, #P7170-1L) staining was used as the loading control; GAPDH was used for all other western blot analyses.

For protein detection and quantification via immunofluorescence, approximately 1.5 × 10^5^ cells were plated in 6-well plates containing 10 mm coverslips under the conditions needed. Then, 4% paraformaldehyde in PBS (pH 7.4) was added for 10 min to fix the cells, and the coverslips were washed (×3) in PBS-T (1× PBS with 0.1% Tween 20), followed by 10 min of permeabilization with PBS containing 0.1% Triton X-100. After washing with PBS-T, the coverslips were blocked in 5% milk for 1 h (RT), and primary antibodies (1:500, Anti-DDDDK tag (binding to the p21-FLAG^®^ tag sequence) antibody, Abcam, #ab205606; 1:500, CDKN1A/p21, Santa Cruz Biotechnology, SCBT #sc6246; 1:200, Histone H2AX, Upstate, #05636-I; 1:200, CDKN1A/p21, Abcam, #ab109520) were incubated overnight at 4°C. The secondary antibodies (IgG Alexa mouse 488, 1:1000, Thermo Fisher Scientific Inc., #A11001 and IgG Alexa 649 rabbit, 1:1000, Thermo Fisher Scientific, #A21245) were added for 1 h at RT in the dark, and mounting media containing DAPI (SlowfadeTM Gold antifade reagent, Thermo Fisher Scientific, #S36938) was added. Fluorescence images were taken with a Leica DM IRB confocal microscope (Wetzlar, #DM6000) and analysed via ImageJ (Version 1.53t, NIH).

### 2.3 Gene Expression Analysis

RNA was extracted using a total RNA isolation kit (NZYtech, #MB13402) according to the manufacturer’s instructions. Complementary DNA (cDNA) was prepared using the cDNA Kit (Applied Biosystems, #4368814) with 200 ng of mRNA per reaction. qPCR reactions (20 μL) contained 10 μL SYBR Green qPCR Supermix (Bio-Rad #1725272), 7 μL of H_2_O, 0.5 μL of each primer, and 2 μL of cDNA were performed in a Bio-Rad CFX96 qPCR machine (Bio-Rad) Initial denaturation was performed at 95°C for 2 min, followed by 40 cycles of 5 s of 95°C denaturation and 30 s of 60°C annealing and extension. A melt curve analysis was then performed with a 5 s hold at 95°C, a 5 s hold at 65°C, and a gradual temperature increase of 0.5°C per cycle up to 95°C. The data were normalized to those of GAPDH and are presented as the means +/− SEMs. The results were analysed via the 2^−ΔΔCT^ method. The primers used in this study are listed in Supplementary Table S1.

### 2.4 Chromatin Immunoprecipitation (ChIP)

Chromatin binding was assessed by chromatin immunoprecipitation (ChIP). Briefly, 7–10 × 10^6^ cells were cultured in T175 flasks and fixed at 80% confluency with 37% formaldehyde in a final concentration of 1%. After collection, cell pellets were diluted in IP buffer (150 mM NaCl, 50 mM Tris-HCl (pH 7.5), 5 mM EDTA, NP-40 (0.5% vol/vol), and Triton X-100 (1.0% vol/vol)) containing protease inhibitors (Roche, Basel, Switzerland #05056489001), and were subsequently lysed with a type A pestle. The pellets were subsequently resuspended in 500 μL of IP buffer and sonicated with a Bioruptor^®^ Pico sonicator (Diagenode, Seraing, Belgium) on high for 7 min, 30 s on, 30 s off. After sonication, samples were centrifuged at 15,000× *g* at 4°C for 10 min, and the supernatant containing the sonicated chromatin was collected. A total of 25 μL was removed for input preparation and chromatin quantification.

Briefly, 175 μL of TE buffer (Tris-HCl [10 mM, pH 8.0], EDTA [1 mM]) and 10 μg/μL RNase A were added. The samples were incubated at 37°C for 30 min, and 10 μg/μL proteinase K was added before they were incubated at 55°C for 30 min and then at 80°C for 2 h. An equal volume of phenol:chloroform:isoamyl alcohol (25:24:1) was added, the samples were then centrifuged at 12,000 rpm for 5 min, and the aqueous phase was transferred to a new tube. The samples were then incubated overnight with a 1/10 volume of NaAc and 2.5 volumes of 100% EtOH. Finally, samples were centrifuged, and the pellet was washed with 70% EtOH. The pellet was resuspended in 25 μL of TE buffer, and the DNA concentration was measured at 260 nm using Nanodrop.

For immunoprecipitation, 10 μg of sonicated chromatin was incubated with 2 μg of CDKNA/p21 antibody (Santa Cruz Biotechnology, #sc6246 or Abcam, #ab18209) or IgG overnight at 4°C. The precipitated chromatin was collected via magnetic beads (Dynabeads^™^ Protein G, Thermo Fisher Scientific Inc., #10004D) and eluted with an elution buffer containing 100 mM NaHCO_3_ and 1% SDS. The primers used for ChIP–qPCR are listed in Supplementary Table S1 and represented as fold enrichment.

### 2.5 Cell Cycle Analysis

For cell cycle stage quantification, cells were trypsinized, collected with a solution containing propidium iodide (PI) (10% Triton X–100, 10 mg/mL RNase A, 1 mg/mL PI, 20% FBS, and PBS), and incubated for 10 min on ice in the dark (200 μL of staining solution/1.5 × 10^5^ cells). Staining with propidium iodide was detected using flow cytometry using the phycoerythrin-Alexa Fluor (PE-A) channel. The results were analysed via the CytExpert program. To synchronize them, cells were plated into 6-well plates at a concentration of 150,000 cells/well in white DMEM (Thermo Fisher Scientific Inc., #31053028) supplemented with 10% charcoal-stripped FBS (Gibco^™^, Thermo Fisher Scientific #12676029) (Day 0). After 48 h (day 2), the media was removed, and a new medium supplemented with no FBS was added for 16 h. The cells were subsequently returned to normal high-glucose DMEM supplemented with 10% FBS (Thermo Fisher Scientific Inc., #17479633), 1% penicillin/streptomycin (Sigma-Aldrich, Merck Group, #P0781), and 1% GlutaMAX (Thermo Fisher Scientific #X0551-100) (day 4). At days 3, 4, 5, and 6, the percentage of cells in each phase of the cell cycle was quantified via fluorescence-activated cell sorting (FACS) as described.

### 2.6 Live-Dead Assay

The percentage of living and dead cells was analyzed using the LIVE/DEAD^™^ Viability/Cytotoxicity Kit for mammalian cells (Thermo Fisher Scientific Inc., #L3224) according to the manufacturer’s instructions modified for use in 3D spheroid culture. Briefly, the mix of 5 μL calcein AM and 30 μL ethidium homodimer-1 was diluted in 10 mL DMEM white media (Thermo Fisher Scientific Inc., #31053028), and then 100 μL was added to each well of a 96-well V-bottom low-adherent plate where the spheroids were grown for 2 days. After 1 h of incubation, images of GFP and RFP signals were taken using the Invitrogen^™^ EVOS^™^ M5000 Imaging System. ImageJ (Version 1.53t, NIH) software was used for the analysis. The total GFP and RFP signal was measured after removing the background signal in each channel, and then the percentage was calculated by dividing each signal by the sum of both channels.

### 2.7 Apoptosis Detection

Apoptosis was measured via an Annexin V-FITC Kit (Beckman Coulter Life Sciences, Brea, CA, USA #IM3546). Briefly, the 1 × 10^5^ cells were stained with 5 μL Annexin V-FITC and 5 μL propidium iodide (PI, 50 μg/mL) for 15 min in the dark. The stain was detected via flow cytometry in the Cytoflex S flow cytometer (Beckman Coulter Life Sciences, #B75442). Annexin V-positive cells were detected via a fluorescein isothiocyanate-Alexa Fluor (FITC-A) laser, and cells positive for propidium iodide were detected via a phycoerythrin-Alexa Fluor (PE-A) laser. The results were analysed via the CytExpert program.

### 2.8 3-(4,5-Dimethylthiazol-2-yl)-2,5-Diphenyltetrazolium Bromide Assay (MTT) Assay

Cell viability was measured by plating cells in a 96-well plate at a concentration of 10,000 cells per well. Following 300 μM H_2_O_2_ treatment, viability was measured 6 and 24 h after with MTT (Thermo Fisher Scientific Inc., #cat. 11312727). Briefly, 20 μL MTT was added at a final concentration of 0.2 mg/mL and incubated for 3 h at 37°C. Resulting crystals were resuspended in DMSO, and the absorption was measured at 570 nm on BioTek Synergy H1 Multimode Reader (Agilent, Santa Clara, CA, USA, #cat. 238261).

### 2.9 Database Analysis

Raw data CEL files and normalised expression matrices from the GSE25066 dataset from the Gene Expression Omnibus (GEO: https://www.ncbi.nlm.nih.gov/gds) were analysed for CDKN1A/p21 expression. Patients were stratified on the basis of their CDKN1A/p21 expression in the CDKN1A/p21 UP group (25% with the highest CDKN1A/p21 expression) and the CDKN1A/p21 DOWN group (25% with the lowest CDKN1A/p21 expression) to maximize biological contrast between expression extremes. To ensure that the observed trends were not dependent on the threshold we examined additional stratifications including middle-range and quartile-based analyses, which shows consistent trends. The pathological complete response (pCR) and distant recurrence-free survival (DRFS) were examined. The percentages of patients in each group with complete pathological response and recurrence-free survival (DRFS = 1) were plotted via GraphPad Prism v10 (GraphPad Software, San Diego, CA, USA). DRFS was analyzed separately using Kaplan–Meier survival curve.

Spatial transcriptomics data from breast cancer tissue (dataset GSE203612 sample: GSM6177603) were analysed via the Crost online platform (https://ngdc.cncb.ac.cn/crost/home) [40]. We used the predefined spatial clusters provided by the original dataset, in which the tissue section was clustered on the basis of gene expression in 8 clusters, and spatial feature plots for candidate genes were generated to visualize the spatial distribution of gene expression across clusters. Gene expression comparisons between normal and tumour tissues of breast cancer patients were performed via the GEPIA2 online platform (http://gepia2.cancer-pku.cn/#index) [41], which utilizes RNA-seq data from TCGA and GTEx. Differential expression was assessed using |log_2_ fold change| ≥ 1 and *p*-value < 0.01 based on log_2_(TPM + 1) expression values.

### 2.10 Statistics

The data are presented as means ± SEMs. Differences between the two groups were assessed using an unpaired two-tailed Student’s *t*-test, unless stated otherwise. For multi-group comparisons, statistical significance was determined using two-way ANOVA followed by Tukey’s multiple-comparison test. Statistical significance was determined for *p*-values of <0.05. For analyses of pathological complete response (pCR) and event incidence (DRFS = 1), group comparisons were performed using Fisher’s exact test (two-tailed). Distant recurrence-free survival (DRFS) was analysed using Kaplan–Meier survival curve, with differences between groups assessed by the log-rank (Mantel–Cox) test. All analyses were performed using GraphPad Prism v10 (GraphPad Software, San Diego, CA, USA).

## 3 Results

### 3.1 Clinical Significance of CDKN1A/p21 Expression in Breast Cancer Patients

**Figure 1:**
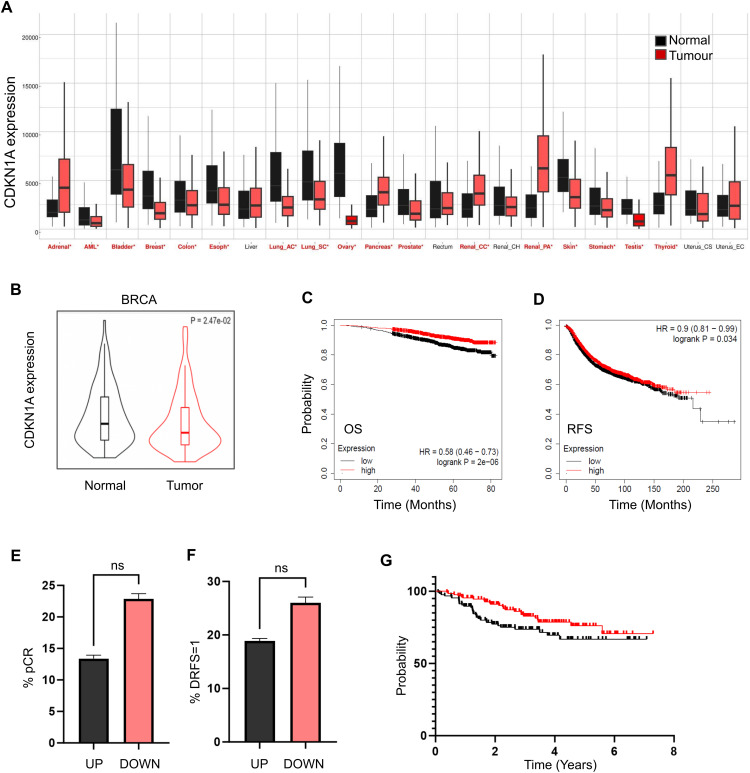
CDKN1A/p21 expression in cancer and its correlation with survival and response to chemotherapy. (**A**). Pancancer analysis of CDKN1A/p21 gene expression in normal (black) or cancer (red) tissue analysed using Tumor, Node, Metastasis (TNM) plot (significant differences according to the Mann–Whitney test are marked with red, *p < 0.05, 15,648 normal, 40,442 tumour, and 848 metastasis samples). (**B**). CDKN1A/p21 mRNA expression in paired tumour and adjacent normal tissues of invasive breast carcinoma was generated with a TNM plot (*p* = 2.47 × 10^−02^), 242 normal, 7569 tumour. Kaplan Meier survival curves of breast cancer patients stratified by CDKN1A/p21 mRNA expression (black = low CDKN1A/p21 expression, red = high CDKN1A/p21 expression). The *Y*-axis represents the probability of survival, and the *X*-axis represents time. (**C**). Overall survival (n = 2976, *p* = 2 × 10^−06^). (**D**). Recurrence-free survival (n = 4929, *p* = 3.4 × 10^−02^). Comparison of the proportions of patients who achieved complete pathological response (pCR), *p* = 0.103 (**E**) and presented with recurrence or death (DRFS = 1) (*p* = 0.23) (**F**) between the high CDKN1A/p21 expression (UP) and low CDKN1A/p21 expression (DOWN) groups. (**G**). Disease-free recurrence survival curve of patients with high CDKN1A/p21 expression (UP) in red and low CDKN1A/p21 expression (DOWN) in black (*p* = 4.72 × 10^−02^). Ns, no significance.

Analysis of pan-cancer patient expression datasets shows that the mRNA levels of CDKN1A/p21 in tumour versus normal tissues differ depending on the tumour type considered. For example, CDKN1A/p21 levels are significantly higher (*p* < 5 × 10^−03^) in normal vs. tumour tissue in bladder, breast, colon, esophagus, lung, ovary, prostate, skin, stomach, and testis, in contrast to observed elevated levels in tumour vs. normal in adrenal, pancreas, renal and thyroid cancers (Fig. 1A and Supplementary Table S2). With respect to breast cancer, regardless of the luminal subtype, CDKN1A/p21 expression is decreased (*p* = 2.47 × 10^−02^) in tumour vs. paired normal adjacent tissue (Fig. 1B), and there is a significant correlation between low levels of *CDKNA/p21* and both poor overall survival (OS) and recurrence-free survival (RFS) (*p* = 2 × 10^−06^ and *p* = 3.4 × 10^−02^, respectively; Fig. 1C,D). These findings suggest that changes in *CDKN1A/p21* may play a role in tumour development and patient response in breast cancer.

To determine whether CDKN1A/p21 expression may be predictive of chemotherapeutic response in breast cancer patients, patients were first stratified on the basis of elevated (UP) or decreased (DOWN) levels of CDKN1A/p21, and response and survival following neoadjuvant taxane–anthracycline chemotherapy were analysed. Patients in the UP group had a lower rate (13.3%) of both complete pathological response (pCR) and recurrence or death events (18.8%) compared with the DOWN group (22.8% and 25.9.%, respectively), although this shows a clear trend the difference did not reach statistical significance (Fig. 1E,F). In addition, patients in the UP group had a significantly greater probability of disease-free recurrence survival (*p* = 4.72 × 10^−02^, Fig. 1G), suggesting that high CDKN1A/p21 levels before therapy are predictive of increased survival in breast cancer patients.

### 3.2 CDKN1A/p21 KD Impairs Spheroid Formation in Breast Cancer Cell Lines

**Figure 2:**
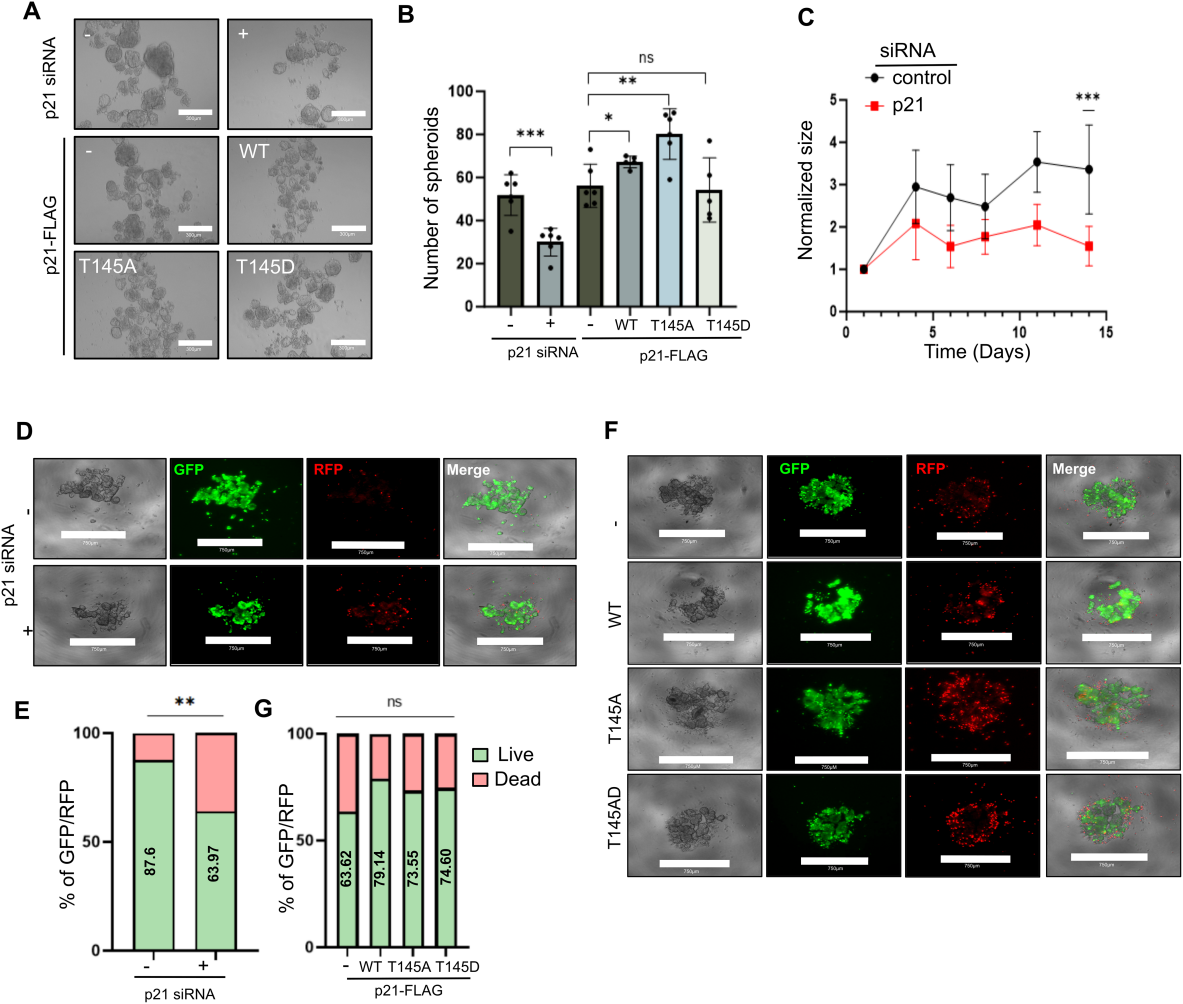
Effects of manipulating CDKN1A/p21 levels on spheroid formation and growth. (**A**). Representative images, scale bar = 300 μm, magnification: 4×. (**B**). Number analysis of spheroids 14 days after plating in a 96-well low-adherence plate for CDKN1A/p21 knockdown (KD) cells or cells overexpressing the p21-FLAG constructs. (**C**). Spheroid size at days 1, 4, 6, 8, 11, and 14 normalized to that on day 1 for CDKN1A/p21-KD cells. Representative images of the live–dead assay (**D**) and bar graph representing the mean percentage of GFP and RFP signals (**E**) in spheroids grown for 2 days in CDKN1A/p21 KD cells, Representative images of the live–dead assay (**F**) and bar graph representing the mean percentage of GFP and RFP signals (**G**) in spheroids overexpressing the p21-FLAG constructs. ((**D**,**F**): Scale bar = 750 μm, magnification = 4×). Ns, no significance. *p < 0.05, **p < 0.01, ***p < 0.001.

As mentioned previously, breast cancer stem cells (BCSCs) are thought to be the initiators of metastatic spread and therapeutic resistance. The growth of mammalian breast cancer cell lines in nonadherent conditions as 3D spheroids enriches this subpopulation of cells, as shown previously by global gene expression analysis comparing cells grown in 2D and 3D, therefore providing a validated method to study BCSCs *in vitro* [42,43]. As expected, using this model, we observe a significant increase in the stem cell markers *CD44* and *ALDH1A* compared with cells grown in 2D (Supplementary Fig. S1A,B). To investigate the role of CDKN1A/p21 in BCSCs growth and survival, the levels of CDKN1A/p21 were manipulated in spheroids, either by transient knockdown (KD) using siRNA or by the overexpression of p21-FLAG-tagged proteins [39] (Figs. 2, S2 and S3). In order to increase the relative expression levels of CDKN1A/p21, MCF7 and BT474 cells were transfected with FLAG-tagged expression constructs containing previously characterised CDKN1A/p21 threonine 145 phosphorylation mutants. CDKN1A/p21 -FLAG overexpression constructs containing either wild type CDKN1A/p21 (WT) or mutated CDKN1A/p21 resulted in nuclear (T145A) or cytoplasmic (T145D) CDKN1A/p21 localization. Cytoplasmic and nuclear CDKN1A/p21 have been shown to exhibit different and often contradictory functions in the context of cancer [44]. The p21-FLAG overexpression constructs presented similar total protein and mRNA expression levels, and as expected, p21-FLAG WT and p21-FLAG T145A showed nuclear localisation in contrast to p21-FLAG T145D in the cytoplasm (Supplementary Fig. S2A–C). In addition, no phenotypic or growth changes were observed in 2D cell culture (Supplementary Fig. S2D). Compared with the control, the KD of CDKN1A/p21 resulted in a significant decrease in the ability to form spheroids (*p* = 9 × 10^−04^), whereas the overexpression of the nuclear p21-FLAG WT or p21-FLAG T145A resulted in increased spheroid formation (*p* = 4.16 × 10^−02^ and *p* = 3.4 × 10^−03^, respectively, Fig. 2A,B). Notably, cytoplasmic p21-FLAG T145D overexpression did not significantly affect the number of spheroids. Considering both the increased spheroid formation following nuclear overexpression and the decrease following KD, we hypothesized that CDKN1A/p21 may play a role in BCSCs survival via a nuclear-specific mechanism. In addition, spheroid size was measured over time, and CDKN1A/p21 KD spheroids were significantly smaller (*p* = 3 × 10^−04^), showing no signs of recovery over a longer period of time (Fig. 2C) accompanied with an increase in cell death (Figs. 2D,E and S2E). Overexpression of nuclear or cytoplasmic CDKN1A/p21 did not increase long-term BCSCs growth or cell death (Figs. 2F,G and S2F). The same trend was also observed in the BT474 cell line: CDKN1A/p21 KD cells formed significantly smaller spheroids (*p* < 10^−04^ whereas p21-FLAG WT overexpression led to larger spheroids (*p* = 3.71 × 10^−02^, Supplementary Fig. S3A,B). The data from both cell lines suggest that CDKN1A/p21 may play a positive role in survival and expansion of breast cancer stem cells, as CDKN1A/p21 KD significantly decreases the ability of breast cancer cells to not only initiate spheroids but also grow and proliferate over time.

### 3.3 Cell Cycle Synchronization Does Not Affect Spheroid Formation or Growth

**Figure 3:**
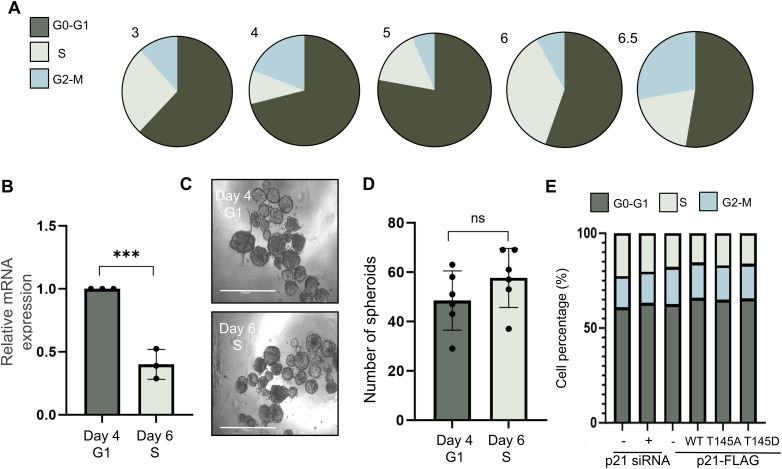
Analysis of the effect of the cell cycle on spheroid initiation. (**A**). Schematic of synchronization after serum deprivation and FACS analysis with PI to determine the percentage of cells in each phase of the cell cycle on various days. The percentage of cells in each phase is presented in pie charts. (**B**). Relative mRNA expression of CDKN1A/p21 determined via qPCR comparing day 4 and day 6, normalized against day 4. (**C**). Representative images of the spheroids formed 14 days after plating either on day 4 (up) of synchronization or on day 6 (down). Scale bar = 750 μm, magnification: 4×. (**D**). Bar graph representing the number of spheroids formed after 14 days. ns = non-significant. (**E**). Bar graph representing the percentage of cells in each phase of the cell cycle 2 days after treatment with CDKN1A/p21 KD or p21-FLAG constructs measured with FACS. n = 3. ns = non-significant, ***p < 0.001.

As CDKN1A/p21 levels vary throughout the cell cycle, peaking during G0–G1 phase and decreasing during S phase [13,14], MCF7 cells were synchronized to enrich for distinct cell cycle populations and therefore populations with varying levels of CDKN1A/p21. Compared with day 6, day 4 was enriched in G1 phase and depleted in S, which was clearly enriched in S phase and depleted in G1 (71.02% vs. 55.4% in G1, and 9.89% vs. 36.58% in S phase, respectively; Figs. 3A and S4). As expected, CDKN1A/p21 mRNA expression was significantly greater on day 4 (G1-enriched, S-depleted) compared to day 6 (S-phase enriched, G1-depleted, *p* = 9.41 × 10^−04^) (Fig. 3B). BCSCs growth was not altered following spheroid initiation in either G1- or S-phase-enriched cells (Fig. 3C,D). In addition, no difference in cell cycle was observed following either KD or overexpression (Fig. 3E), suggesting that the effect we observed on BCSCs (Fig. 2) following CDKN1A/p21 KD or overexpression was not due to alterations in cell cycle progression but rather a more direct effect on cancer stem cell growth and proliferation.

### 3.4 CDKN1A/p21 Promotes the Resistance of 3D Spheroids to Oxidative Damage

**Figure 4:**
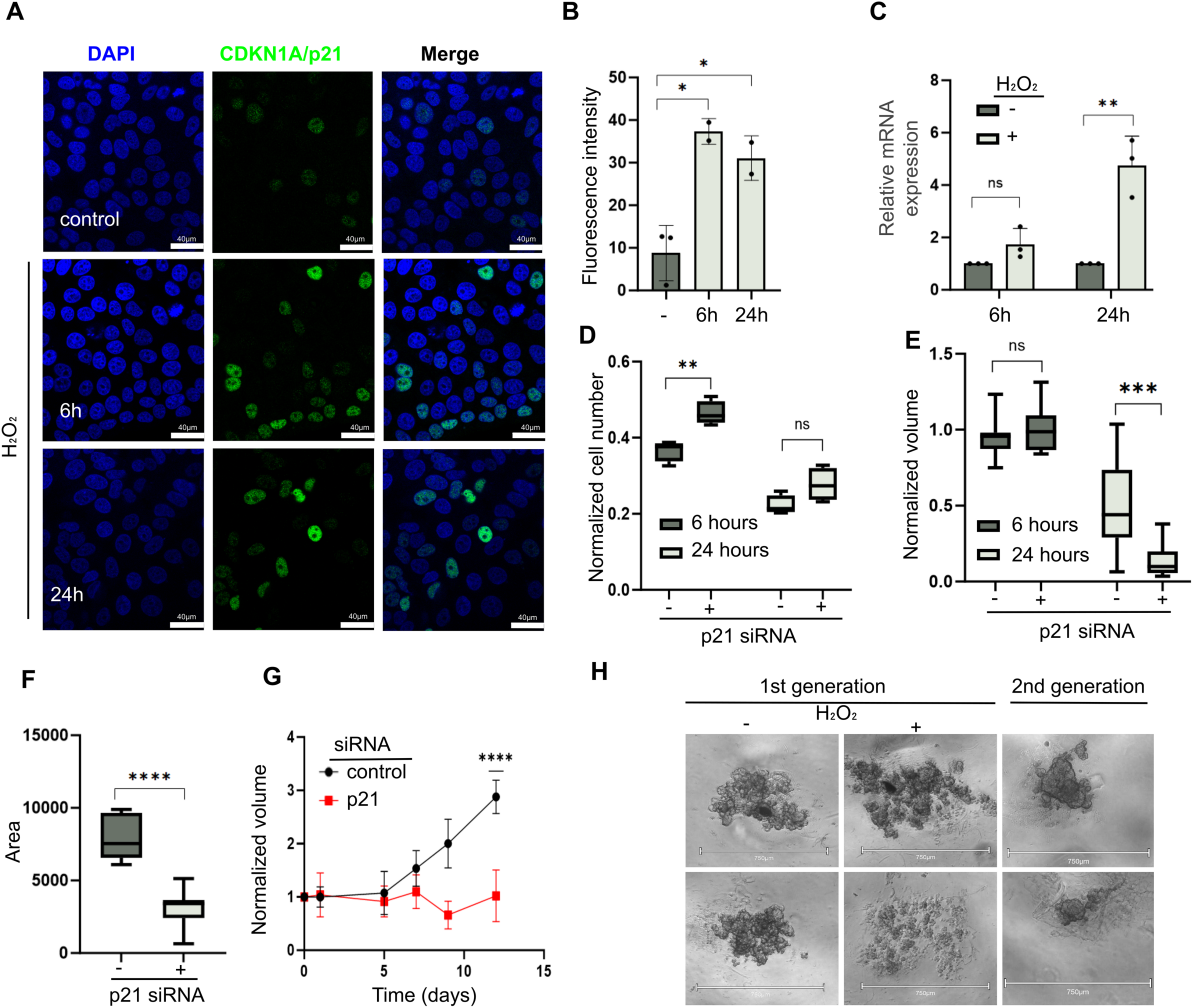
Spheroid growth upon oxidative damage after manipulation of CDKN1A/p21 expression levels. (**A**). Representative images of immunofluorescence analysis of CDKN1A/p21protein levels after treatment with 300 μM H_2_O_2_. Scale bar = 40 μm; magnification = 63×. (**B**). Relative protein levels of CDKN1A/p21 after treatment. (**C**). Relative mRNA expression of CDKN1A/p21 after treatment compared with the control. (**D**). Normalized cell numbers of CDKN1A/p21 KD or siRNA control cells grown in 2D monolayers at 6 h and 24 h after treatment with 300 μM H_2_O_2_. (**E**). Normalized spheroid volume of spheroids originating from CDKN1A/p21 KD or siRNA control cells at 6 h and 24 h after treatment with 300 μM H_2_O_2_. (**F**). Bar graph representing the volume of second-generation spheroids 12 days after plating, comparing CDKN1A/p21 KD or siRNA control-originated spheroids expressed as the measured area in pixels^2^. (**G**). Growth rate of second-generation spheroids. (**H**). Spheroids derived from CDKN1A/p21-KD or siRNA control cells before and after treatment with 300 μM H_2_O_2_ for 6 h, and second-generation spheroids 12 days after plating. Scale bar = 750 μm; magnification = 10×. ns = non-significant, *p < 0.05, **p < 0.01, ***p < 0.001, ****p < 0.0001.

It has been previously shown that CDKN1A/p21 levels increase following H_2_O_2_-induced oxidative damage [45–47]. To study the impact of H_2_O_2_ and DNA damage on our BCSCs model, the cells were exposed to a range of H_2_O_2_ concentrations, and alterations in the cell cycle were measured (Supplementary Fig. S5A–D). Exposure to 300–600 μM H_2_O_2_ for 6 h resulted in an increased percentage of cells in the G2 phase and a decrease in the percentage of cells in the S and G1 phases, while doses of approximately 1000 μM H_2_O_2_ were lethal. The levels of CDKN1A/p21 after H_2_O_2_ were increased at 6 and 24 h of exposure (Figs. 4A–C and S5E, *p* = 1.13 × 10^−02^ and *p* = 2.81 × 10^−02^, respectively). As a control for a functional DNA damage response, the levels of the DNA damage marker phospho-γH2AX [48] were measured and as expected showed an increased following damage (Supplementary Fig. S5F,G). To study the effect of CDKN1A/p21 levels on resistance to DNA damage, CDKN1A/p21 was knocked down, and survival in both 2D monolayers and 3D spheroids after H_2_O_2_ exposure for 6 h was compared. In 2D monolayer culture, cell survival was greater in the CDKN1A/p21 KD group than in the control group at 6 h following H_2_O_2_ exposure (*p* = 3.4 × 10^−03^), whereas 24 h after oxidative damage, no significant difference in survival was detected between the KD and control groups (Fig. 4D). In contrast, in BCSCs, 24 h after H_2_O_2_ exposure, the spheroid volume was significantly reduced following CDKN1A/p21 KD (*p* = 9 × 10^−04^), suggesting increased sensitivity to oxidative damage (Fig. 4E). Similarly, second-generation spheroids, a measure of “stemness,” were significantly smaller in size than the control spheroids when CDKN1A/p21 was knocked down prior to oxidative damage (*p* < 10^−04^) (Fig. 4F–H). In MDA-MB-231 triple-negative breast cancer (TNBC) cells, CDKN1A/p21 mRNA increased after 6 h treatment with H_2_O_2_, however 24 h after H_2_O_2_ exposure the levels returned to basal levels (Supplementary Fig. S6A) in contrast to the increase in CDKN1A/p21 observed in hormone receptor positive MCF7 cells (Fig. 4C). CDKN1A/p21 KD reduced proliferation in 2D culture under basal conditions (Supplementary Fig. S6B), whereas after H_2_O_2_ exposure, CDKN1A/p21 KD cells showed a modest increase in cell number compared to control (Supplementary Fig. S6C,D). In 3D spheroid cultures, CDKN1A/p21 knockdown led to increased spheroid growth under untreated conditions but no significant difference after oxidative stress (Supplementary Fig. S6E–G). Notably, MDA-MB-231 spheroids were more irregular and heterogeneous in size and morphology than the compact, uniform spheroids of HR^+^ lines, which limited quantitative precision and statistical significance (Supplementary Fig. S6H). These findings suggest that CDKN1A/p21 plays a context-dependent role in TNBC, differing from the clearer survival-supporting function observed in HR^+^ breast cancer models. These results suggest that CDKN1A/p21 levels may play a key role in the resistance of 3D HR positive breast cancer spheroids upon H_2_O_2_ treatment.

### 3.5 The CDKN1A/p21 Inhibitor UC2288 Impairs Spheroid Growth but Increases Spheroid Formation

**Figure 5:**
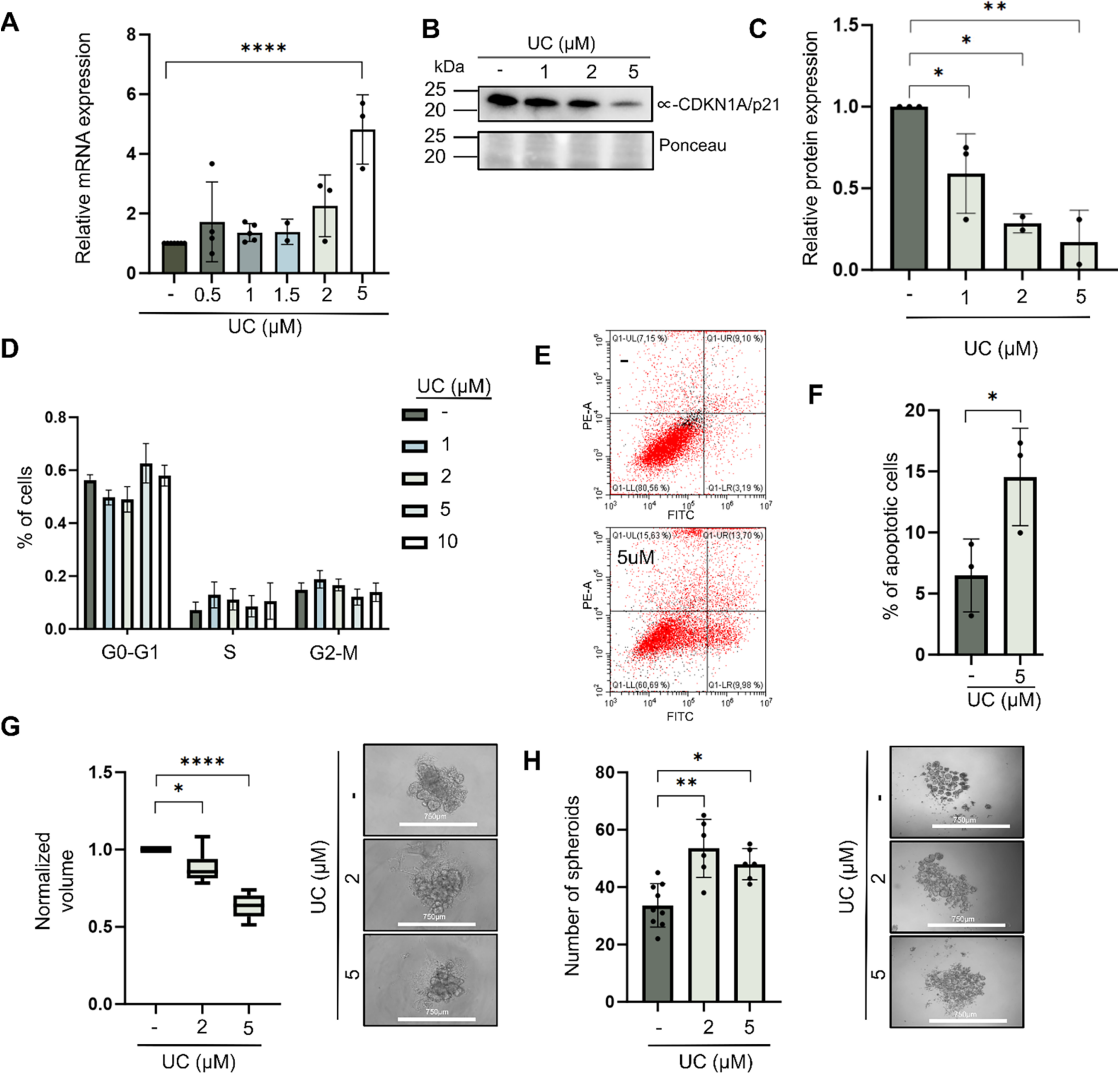
Effects of the CDKN1A/p21 inhibitor UC2288 on CDKN1A/p21-related cell processes and spheroid formation. (**A**). Relative mRNA expression of CDKN1A/p21 after treatment with UC2288 at 0.5, 1, 1.5, 2 μM, and 5 μM compared with the control (DMSO). (**B**). Representative western blot of cells after treatment with UC2288 at 1, 2, or 5 μM showing the levels of DKN1A/p21 using a specific antibody, ponceau staining is shown as a loading control. (**C**). Bar graph of the quantification of CDKN1A/p21 protein expression after treatment with UC2288 at 1, 2, and 5 μM compared with the control (DMSO). (**D**). Bar graph representing the percentage of cells in each phase of the cell cycle after treatment with various concentrations of UC2288 (DMSO, 1 μM, 2 μM, 5 μM, or 10 μM) (n = 3). (**E**). Flow cytometry analysis of the percentage of live/apoptotic/necrotic cells after treatment with 5 μM UC2288 for 3 days with daily treatment. (**F**). Histogram representing the number of apoptotic cells compared with the control (DMSO) (n = 3). (**G**). Representative images and growth analysis of spheroids formed 8 days after culture in the presence of 2 μM or 5 μM UC2288. The data are normalized against the control (DMSO). Scale bar = 750 μm; magnification = 10×. (**H**). Representative images and analysis of the number of spheroids after 14 days with 2 μM and 5 μM UC2288. Scale bar = 750 μm; magnification = 4×. Statistical significance was determined by two-way ANOVA followed by Tukey’s multiple-comparison test. *p < 0.05, **p < 0.01, ****p < 0.0001.

The small molecule inhibitor UC2288 (UC) has been shown previously to reduce the total protein level of CDKN1A/p21 [49] and inhibit tumour formation *in vitro* [50] and *in vivo* in mouse xenografts [51]. MCF7 cells treated with various concentrations of UC2288 presented a significant increase in CDKN1A/p21 mRNA at the highest dose (5 μM) 48 h after treatment (Fig. 5A) and a significant decrease in protein levels (Fig. 5B,C), although no alterations in the cell cycle were observed (Fig. 5D). Treatment with UC2288 also significantly increased nuclear levels of CDKN1A/p21 compared with the control as it was observed with IF (*p* = 6 × 10^−03^; Supplementary Fig. S7A,B). Compared with that of the control, cell viability after UC2288 treatment was measured by determining the proportion of apoptotic cells, which increased following UC228 treatment (*p* = 4.87 × 10^−02^) (Fig. 5E,F). Thus, UC2288 may mediate its action by inhibiting cytoplasmic CDKN1A/p21 without affecting its nuclear function in the cell cycle. The inhibitor significantly reduced the size of spheroids in both MCF7 (Fig. 5G) and BT474 (Supplementary Fig. S3C) cells. However, when MCF7 cells were treated with UC2288 for 2 days and then plated in low-adherence conditions to initiate spheroid formation, the number of spheroids significantly increased after treatment compared with the control (*p* = 2.1 × 10^−03^ and *p* = 1.5 × 10^−02^) (Fig. 5H). The size of the spheroids was also measured at different timepoints (Supplementary Fig. S7C). After treatment with UC2288, the spheroid size slightly but significantly decreased only on day 4 after treatment with the higher concentrations (*p* = 4 × 10^−02^). This data may suggest that CDKN1A/p21 has distinct cytoplasmic and nuclear functions, where inhibition of its cytoplasmic form by UC2288 reduces spheroid growth and increases apoptosis, but paradoxically enhances spheroid initiation—implying a complex role in regulating BCSCs dynamics—and highlights that partial inhibition of CDKN1A/p21 may inadvertently support the survival of resistant stem-like gene expression signatures populations associated with poor prognosis in patients.

### 3.6 CDKN1A/p21-Associated Gene Signature Is Associated with Recurrence and Metastasis in Breast Cancer Patients

**Figure 6:**
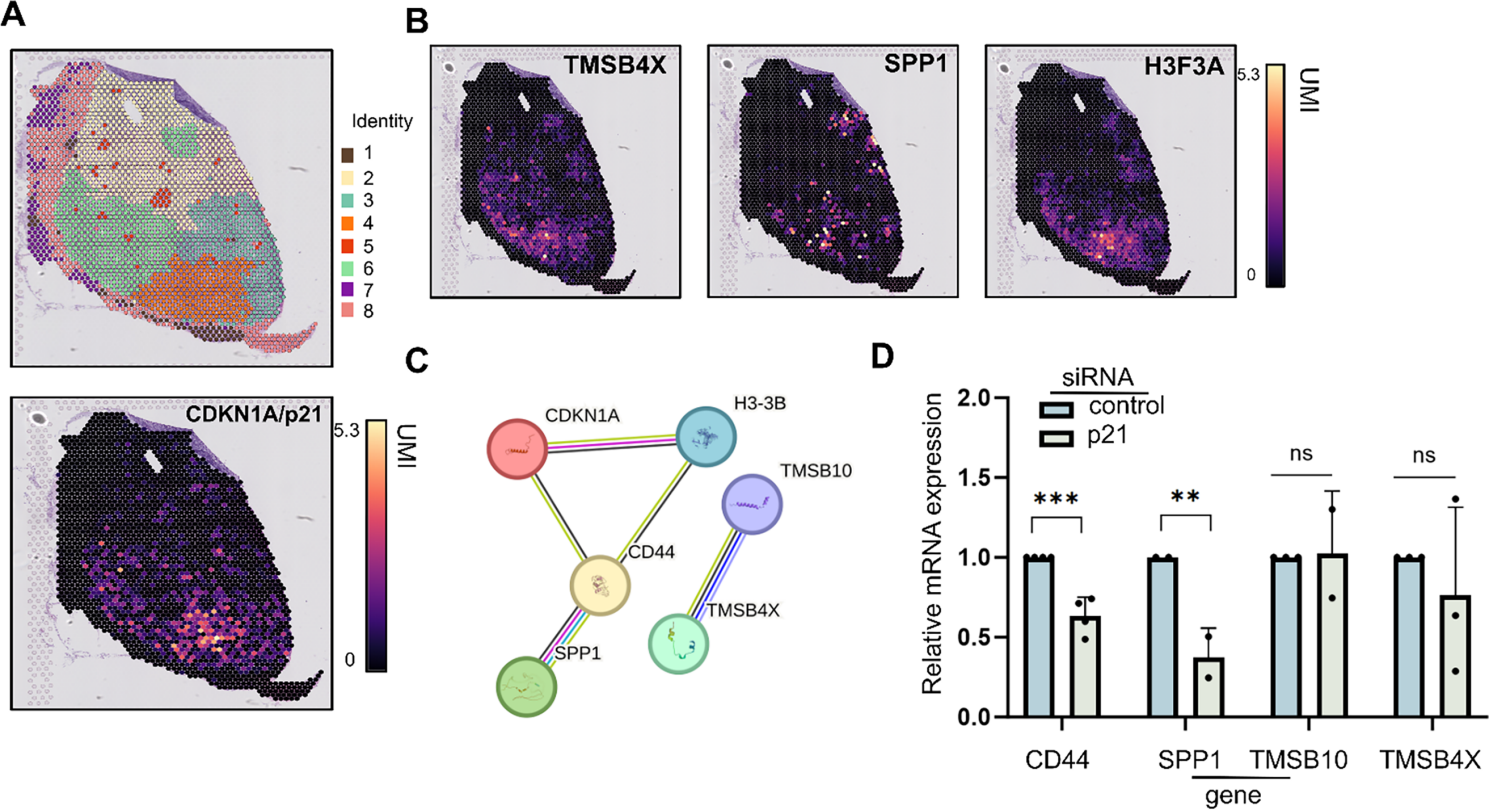

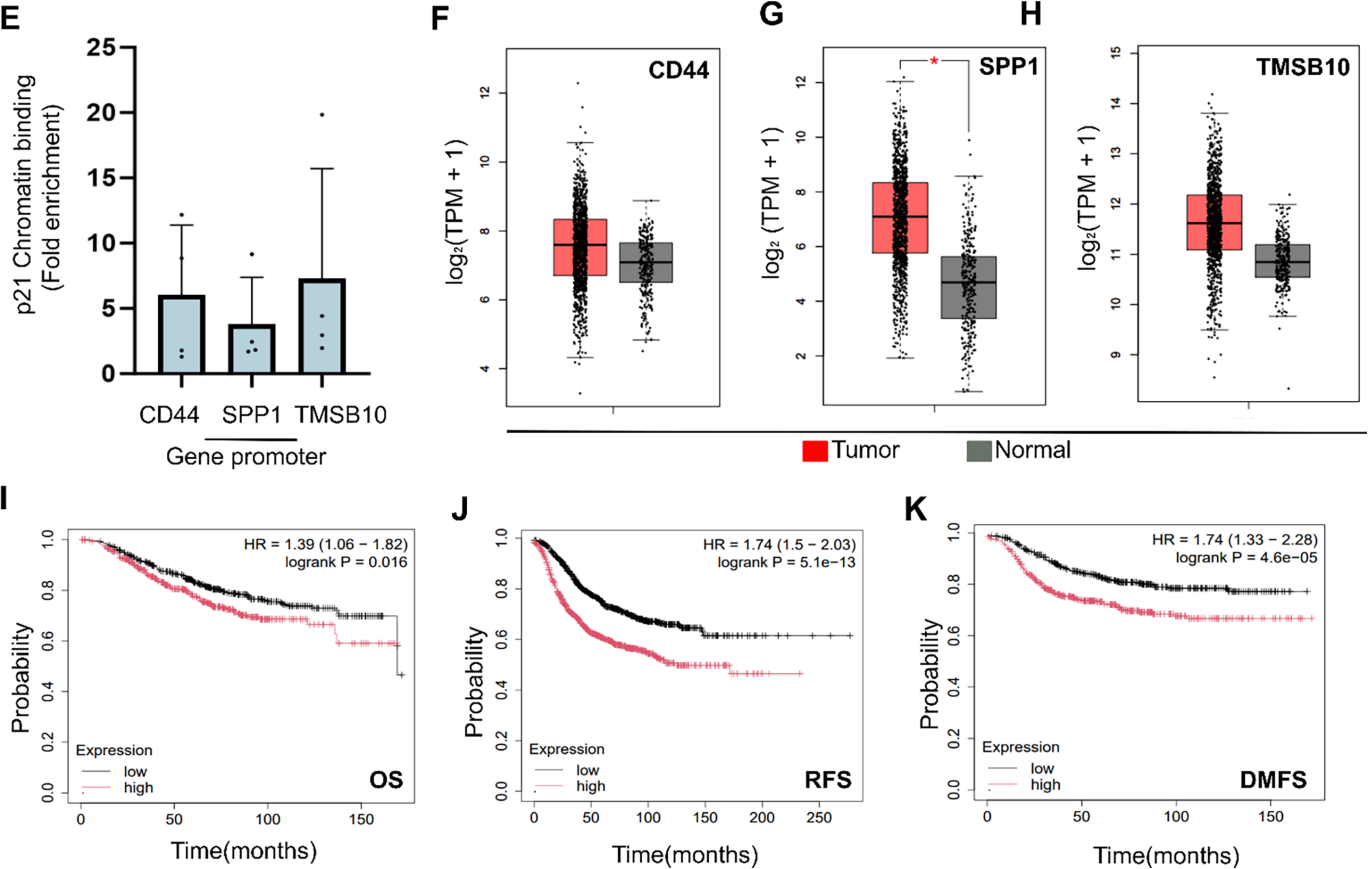
The CDKN1A/p21-associated gene signature is spatially enriched in breast cancer tissue and is associated with poorer cancer patient outcomes. (**A**). Spatial transcriptomics map showing RNA expression-based clustering of breast cancer tissue section of the sample GSM6177603 from Gene Expression Omnibus database [61] (upper panel) and spatial expression plot of CDKN1A/p21 (lower panel) analysed using CROST [40]. Expression values represent log-transformed, library-size-normalized unique molecular identifier (UMI) counts per spatial spot; the scale bar is shown. (**B**). Spatial expression plots of SPP1, TMSB4X, and H3F3A, expression range as (**A**). (**C**). Protein–protein network of predicted or known interactions for candidate genes using the STRING database [62]. (**D**). Relative mRNA expression of CD44, SPP1, TMSB4X, and TMSB10 after treatment with the siRNA against CDKN1A/p21 compared with the siRNA control. ns no significance. (**E**). Chromatin immunoprecipitation quantitative PCR (ChIP–qPCR) reveals CDKN1A/p21-bound at the CD44, SPP1, and TMSB10 proximal promoters, data normalized to the fold enrichment of a control region. Gene expression of CD44 (**F**), SPP1 (**G**), and TMSB10 (**H**) in breast cancer tissue (red) vs. normal (control) (tumour: n = 1085 normal n = 291, BRCA dataset). Kaplan–Meier survival curves of breast cancer patients with combined high (red) or combined low (black) expression levels of CDKN1A, CD44, SPP1, and TMSB10, focusing on (**I**) overall survival (OS), (**J**) recurrence free survival (RFS), and (**K**) distant metastasis-free survival (DMFS). ns = non-significant, *p < 0.05, **p < 0.01, ***p < 0.001.

The effects of CDKN1A/p21 KD on spheroid initiation and resistance to oxidative damage may be mediated by an unknown role of CDKN1A/p21 in nuclear gene regulation. To identify candidate genes that are coregulated with CDKN1A/p21 in a tumour context, we analysed spatial transcriptomics data from the breast cancer tissue dataset GSE203612 [52]. Clustering of the tissue sections revealed the presence of 8 regions with similar transcriptional profiles (Fig. 6A). CDKN1A/p21 expression was highly enriched in cluster 4 (Fig. 6B) along with other enriched genes within the same cluster: SPP1, TMSB4X, TMSB10 and H3F3A (Figs. 6B and S8A). Secreted phosphoprotein 1 (SPP1) is involved in bone mineralization, cell adhesion, migration and the immune response [53–55]. In addition, it is a known interactor of CD44 [56,57], a protein with a central role in breast cancer stem cell biology [38] (Fig. 6C). TMSB4X (thymosin beta-4) and TMSB10 (thymosin beta-10) are proteins that bind to actin and regulate its dynamics. All three genes have been previously shown to promote metastasis in various cancers, such as breast and colorectal cancer [58–60]. In MCF7s CDKN1A/p21 KD cells have significantly lower CD44 (*p* = 7 × 10^−04^) and SPP1 (*p* = 1.4 × 10^−03^) compared to siRNA control cells, while there is no significant change in TMSB4X and TMSB10 expression (Fig. 6D). To investigate whether CDKN1A/p21 regulates the expression of these genes by binding directly to their promoters, ChIP–qPCR analysis was performed using two different CDKN1A/p21 antibodies (Supplementary Fig. S8B). The binding to the promoters of 5 cell cycle-related genes (CCNE2, PLK1, KIF4A, CDK2, WEE1) that CDKN1A/p21 has been previously shown to bind [24] was used as a positive control. We observed clear enrichment of CDKN1A/p21 to the promoter regions of CD44, SPP1, and TMSB10 (Figs. 6E and S8B), suggesting that the loss of expression of these genes in the absence of CDKN1A/p21 may be directly controlled via promoter binding and transcriptional activation.

Analysis of the gene expression levels of CD44, SPP1, and TMSB10 via GEPIA [41] revealed that these genes are overexpressed in breast cancer patients (Fig. 6F–H) and patients that express a combined high expression signature considering CDKN1A/p21, CD44, SPP1, and TMSB10 present a significantly lower probability of OS (Fig. 6I) (*p* = 1.6 × 10^−02^), recurrence-free survival (RFS) (Fig. 6J) (*p* = 5.1 × 10^−13^), and distant metastasis-free survival (DMFS) (Fig. 6K) (*p* = 4.6 × 10^−05^). In addition, this combinational signature was clearly more predictive of a poor patient outcome than single gene stratification (Supplementary Fig. S8C–E).

## 4 Discussion

In this study, we aimed to investigate the role of CDKN1A/p21 in the survival and expansion of breast cancer stem cells. CDKN1A/p21 can have both pro-tumourigenic and tumour suppressor roles in cancer [44], and its expression levels vary between different cancer types and stages [11]. In breast cancer, CDKN1A/p21 expression is significantly decreased in tumour tissues compared to normal, correlating with a poorer survival outcome and increased probability of relapse and metastasis following chemotherapy. High levels of CDKN1A/p21 are predictive of better clinical outcomes, which may be due to its main function in cell cycle arrest [63]. Future clinical data analysis should focus on single-cell expression profiles with the aim of identifying rare subpopulations of cancer cells with high CDKN1A/p21, which may be responsible for cancer reawakening following chemotherapy. KD of CDKN1A/p21 expression reduced spheroid growth and viability in both MCF7 and BT474, while overexpression of total and nucleus-localized CDKN1A/p21 increased spheroid formation ability with no effect on cell viability, suggesting that CDKN1A/p21 may promote breast cancer stem cell survival mainly through a nuclear action. These results suggest that CDKN1A/p21 may play a protective role in breast cancer stem cell survival, as reducing its levels results in fewer spheroids with impaired growth and decreased cell viability. However, overexpression of CDKN1A/p21 failed to produce the opposite effect. A possible reason is that certain threshold levels of CDKN1A/p21 are necessary for cancer stem cell survival, and increasing those thresholds does not result in increased survival.

Although CDKN1A/p21 is generally expressed at lower levels in tumour tissues compared with normal tissues, our findings show that it remains crucial for the survival of breast cancer stem cells (BCSCs), highlighting its context-dependent, sometimes paradoxical roles depending on cell type, localisation, and hormonal environment [11,44]. In HR^+^ breast cancers, nuclear CDKN1A/p21 works downstream of p53 and ERα to halt the cell cycle and maintain genomic stability, which helps explain why its expression drops in more advanced tumours where these pathways are lost. In contrast, in BCSCs and stressed tumour cells, CDKN1A/p21 can be activated independently of p53, helping cells survive under oxidative or therapeutic stress [15]. Our experiments in MDA-MB-231 triple-negative cells further underscore how CDKN1A/p21’s function depends on both receptor status and cellular context: unlike HR^+^ models (MCF7, BT474), where CDKN1A/p21 collaborates with p53 and ERα to promote stemness and oxidative stress resistance, TNBC cells lack ER signalling and often carry p53 mutations, which may disconnect CDKN1A/p21 from its usual nuclear regulatory network. As a result, depleting CDKN1A/p21 in MDA-MB-231 cells can activate compensatory pathways that partially sustain proliferation under oxidative stress, consistent with the modest increase in viability we observed after H_2_O_2_ treatment. In 3D cultures, while basal loss of CDKN1A/p21 encouraged spheroid growth, oxidative stress largely erased these differences, reflecting the more variable redox responses in TNBC. The irregular morphology and heterogeneity of MDA-MB-231 spheroids also make quantitative comparisons with the more uniform HR^+^ spheroids challenging. Overall, our data suggest that although CDKN1A/p21 supports oxidative adaptation in both breast cancer subtypes, its impact is heavily influenced by hormonal and genetic context, with HR^+^ cells depending more on CDKN1A/p21-driven pathways for survival under stress than TNBC cells. Future studies will aim to understand this dependence in more detail in HR^+^ and HR^−^ cell lines and patient-derived cultures, in addition to a more in-depth analysis of the interacting partners and the mechanism of chromatin recruitment, regulation, and BSCS gene expression changes genome-wide.

In addition, we found that G1 phase enriched populations exhibit similar spheroid formation compared with S phase enriched populations, regardless of their different levels of CDKN1A/p21 expression, implying that the effect of CDKN1A/p21 KD on spheroid initiation ability is not via increased cell number in G0–G1, but rather reflects a cell-specific effect, possibly through the regulation of gene expression in a niche population of cancer stem cells. Further experiments are needed to confirm whether cells permanently resting in the G0 phase in the quiescent state have a survival advantage. Even in the absence of damage, cancer stem cells may remain in a protective quiescent state without cycling [64], and the levels of CDKN1A/p21, may be important in sustaining this state. Recently, it has been shown that knockout of CDKN1A/p21 in non-small cell lung cancer cell lines leads to a reduction in CDKN1A/p21-dependent quiescence and results in reduced survival after chemotherapy [65].

It has been proposed that after chemotherapeutic damage, BCSCs survive and enter a senescent state, eventually leading to relapse and metastasis [66–68]. Senescence has been correlated with high levels of CDKN1A/p21 [69–72]; therefore, we hypothesized that CDKN1A/p21 may play a role in the survival of these cells upon DNA damage. We demonstrated that cells cultured in 2D and 3D conditions respond differently to DNA damage caused by oxidative stress. In 2D, CDKN1A/p21 KD resulted in a transient survival advantage compared to control, but long-term growth was inhibited in both conditions. In contrast, CDKN1A/p21 KD spheroids reduced survival after damage and impaired second-generation growth. Compared with 2D-cultured cells, 3D-cultured cells exhibit increased expression of stem cell markers [43] and greater resistance to chemotherapy [73,74]. The differences observed in the response of 2D cells and 3D spheroids after oxidative damage suggest distinct roles and actions of CDKN1A/p21 in breast cancer stem cells and in normal breast cancer cells.

To our knowledge, UC2288 is the only CDKN1A/p21 protein inhibitor that has been shown to have an antitumour function in preclinical research, both *in vitro* [50] and *in vivo* [51]. Indeed, continued UC2288 treatment reduced spheroid growth, while UC2288 reduced total protein levels of CDKN1A/p21, it failed to maintain a reduction in mRNA expression, suggesting the action is mediated via alterations in protein turnover. Moreover, the cell cycle was not altered even though apoptosis was increased, which may suggest that UC2288 cannot inhibit nuclear CDKN1A/p21. Notably, UC2288 treatment increased spheroid formation, indicating that it may promote BCSCs survival. Therefore, developing new small molecules that inhibit nuclear CDKN1A/p21 is necessary for unraveling its therapeutic potential in breast cancer.

Spatial transcriptomic analysis of breast cancer tissue revealed CDKN1A/p21 clusters with SPP1, TMSB4X, TMSB10, and H3F3A. Immunohistochemical analysis (IHC) has previously revealed that high TMSB10 levels in breast cancer patients predict poor prognosis and distant metastasis, whereas silencing TMSB10 in breast cancer cells reduces the proliferation, migration, and invasion of breast cancer cells both *in vitro* and *in vivo* in mouse xenographs [58]. Elevated SPP1 levels in plasma have also been shown to be associated with a poor outcome for breast cancer patients [75], and silencing SPP1 in breast cancer cells reduces spheroid initiation ability, indicating that SPP1 is involved in breast cancer stem cell survival [76]. In addition, high SPP1 mRNA levels have been shown to predict resistance to tamoxifen in breast cancer patients [77], and it has been demonstrated *in vivo* in mice that activation of the JNK pathway after chemotherapy promotes lung metastasis through the upregulation of SPP1 [78]. Moreover, single-cell RNA sequencing in a cohort of triple-negative breast cancer (TNBC) patients revealed that, in the chemotherapy-resistant group, there was upregulation of macrophage-secreted SPP1, which interacts with CD44 in cancer cells [57]. In renal cell carcinoma (RCC), single-cell patient data have shown that SPP1 secreted from cancer cells interacts with CD44-positive T cells, promoting resistance to immune checkpoint inhibitors (ICIs) [79]. Therefore, SPP1 may promote breast cancer stem cell survival and chemoresistance through interaction with CD44. CD44 is well established in breast cancer stem cell biology [38] and is known to promote the survival of breast cancer stem cells after treatment [80,81]. We found that upon CDKN1A/p21 KD, the relative mRNA expression of these key drivers of chemoresistance and metastasis was significantly decreased, directly mediated by CDKN1A/p21 promoter binding. CD44, SPP1, and TMSB10 expression are increased in the tissues of BRCA patients compared with normal tissues, while patients with combined high expression of these genes and CDKN1A/p21 have lower probabilities of overall, recurrence-free, and distant metastasis-free survival. These findings may indicate that the nuclear chromatin binding action of CDKN1A/p21 may possibly play a role in therapy resistance and metastatic potential in patients by regulating the expression of a combinational gene signature driving a more aggressive disease. Limitations of this study include the use of a small number of gene loci for CDKN1A/p21 promoter binding and a focus on potential driver genes identified by the spatial transcriptomics. In future we aim to address these limitations by performing global chromatin binding analysis (ChIP-seq) in breast cancer cells lines and patient derived cultures comparing to the global gene expression signature (RNA-seq) observed in the presence or absence of CDKN1A/p21 after DNA damage in order to verify our findings in a more general scale and gain insight into the pathways and larger scale chromatin alterations that may take place.

In summary, even though nuclear CDKN1A/p21 is considered to have a tumour suppressive function, its role in regulating gene expression may actually promote the survival of the breast cancer stem cell niche, both under basal conditions and after oxidative stress. This opens the way for new therapeutic strategies, inhibiting this nuclear action in combination with chemotherapy to improve patient outcomes in advanced disease.

## 5 Conclusions

We propose a novel function for CDKN1A/p21 in contributing to the survival of BCSCs under basal conditions and following oxidative damage. Mechanistically, we suggest that CDKN1A/p21 directly regulates gene expression via chromatin binding to the promoters of genes related to the survival of breast cancer stem cells (BCSCs). Therefore, targeting CDKN1A/p21 in combination with chemotherapy could offer a promising therapeutic pathway for eliminating breast cancer stem cells and improving treatment efficacy.

## Supplementary Materials



















## Data Availability

All additional data and materials are available in the supplementary materials.

## Supplementary Materials

The supplementary material is available online at https://www.techscience.com/doi/10.32604/or.2026.074965/s1.

## Abbreviations

**Abbreviation**: **Full Form**
BCSCs: Breast cancer stem cells
CDKN1A/p21: Cyclin-dependent kinase inhibitor 1A/p21
qPCR: Quantitative polymerase chain reaction
EMT: Epithelial–mesenchymal transition
TF: Transcription factor
DMEM: Dulbecco’s modified eagle medium
FBS: Fetal bovine serum
P/S: Penicillin/Streptomycin
EGF: Epidermal growth factor
PBS: Phosphate-buffered saline
DAPI: 4^′^,6-diamidino-2-phenylindole
ChIP: Chromatin immunoprecipitation
FACS: Fluorescence-activated cell sorting
MTT: 3-(4,5-dimethylthiazol-2-yl)-2,5-diphenyltetrazolium bromide
GEO: Gene expression omnibus
TCGA: The cancer genome atlas
GTEx: Genotype-tissue expression
OS: Overall survival
RFS: Recurrence-free survival
DMFS: Distant metastasis-free survival
PE-A: Phycoerythrin-Alexa Fluor
FITC-A: Fluorescein isothiocyanate-Alexa Fluor
EtOH: Ethanol
SDS-PAGE: Sodium dodecyl sulfate-polyacrylamide gel electrophoresis
DMSO: Dimethyl sulfoxide
IHC: Immunohistochemistry
KD: Knockdown
UC: UC2288
