# Supplementary material for: CDKN1A/p21 Influences the Survival and Expansion of Breast Cancer Stem Cells after Oxidative Damage
Source: Oncol Res. 2026 Apr 22;34(5):20. doi: 10.32604/or.2026.074965 (PMC13126419; doi:10.32604/or.2026.074965)
Supplement: Supplementary file 9 [file OncolRes-34-74965-s009.docx]

**
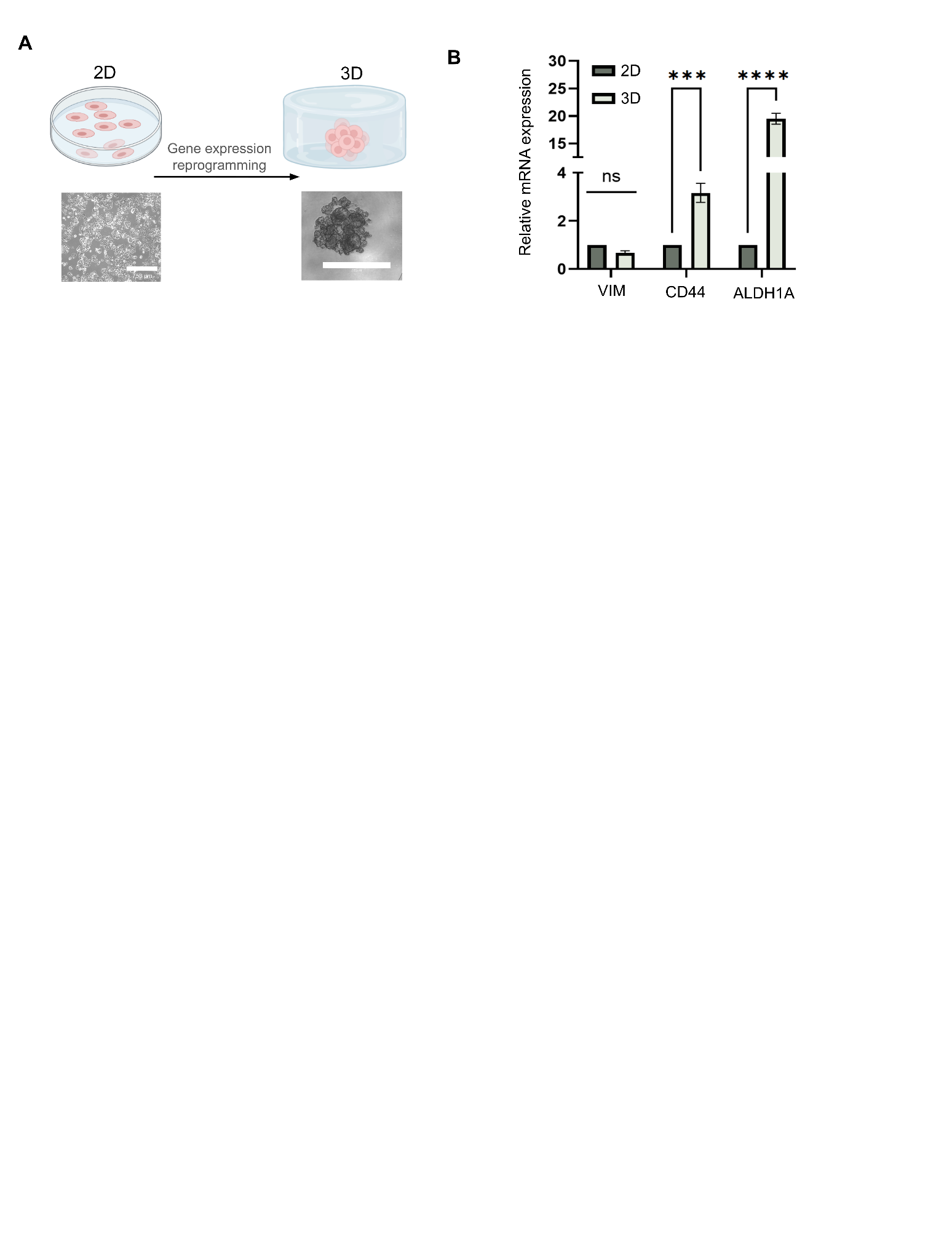
**

**Supplementary Figure 1: Enrichment in BCSCs markers through growing in non-adherent conditions. A. Illustration and brightfield images of MCF7 cells growing in 2D or 3D non-adherent conditions. Left image: Scale bar = 750 μm; magnification = 4× Right image: Scale bar = 750 μm; magnification = 10×) .B. Relative mRNA expression of *Vimentin*, *CD44*, and *ALDH1A* of MCF7 cells growing in 2D or 3D non-adherent conditions (ns, no significance. *p*>0.05, ****p*<0.001, *****p*<0.0001).**

**
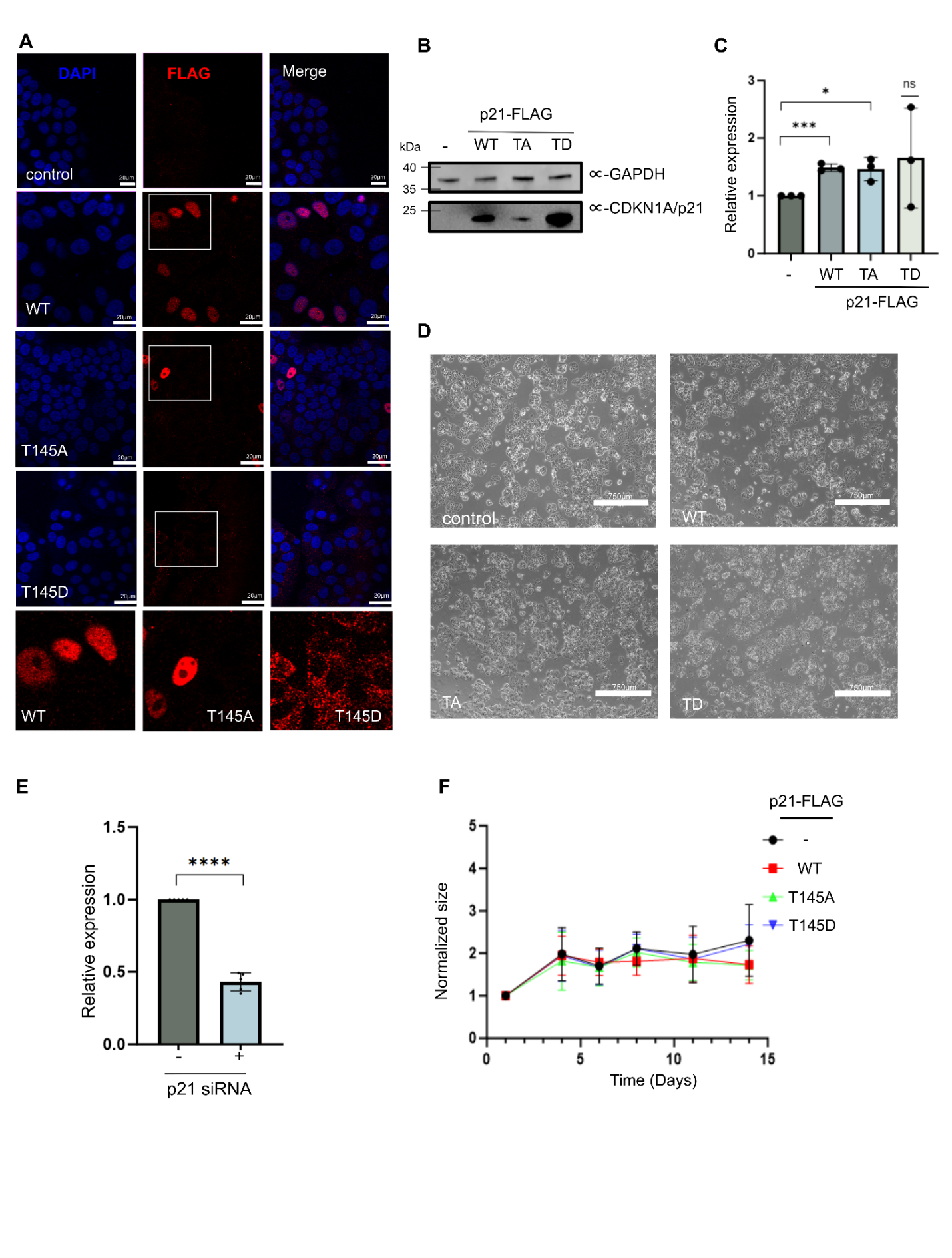
**

**Supplementary Fig. 2: Transfection with the Flag constructs for CDKN1A/p21 overexpression**

**A. Immunofluorescence analysis of the protein levels of DDDDK (FLAG) tag after overexpression of the FLAG constructs (Anti-DDDDK tag-Red, DAPI-Blue). Scale bar = 20 µm. Magnification: 63× B. Western blot analysis of the protein levels of CDKN1A/p21 after p21-FLAG constructs overexpression, with GAPDH used as a loading control. C. Relative mRNA expression of CDKN1A/p21 48 hours after transfection with p21-FLAG constructs. n=3. D. Brightfield images of the cells 48h after transfection p21-FLAG constructs. Scale bar = 750 µm. Magnification: 4×. E. Relative mRNA expression of CDKN1A/p21 knockdown cells 48 hours after transfection compared with control siRNA. F. Spheroid size at day 1,4,6,8,11, and 14 normalized with day 1 for p21-FLAG overexpressing cells. Ns = non significant, **p*<0.05, ****p*<0.001, *****p*<0.0001**

**
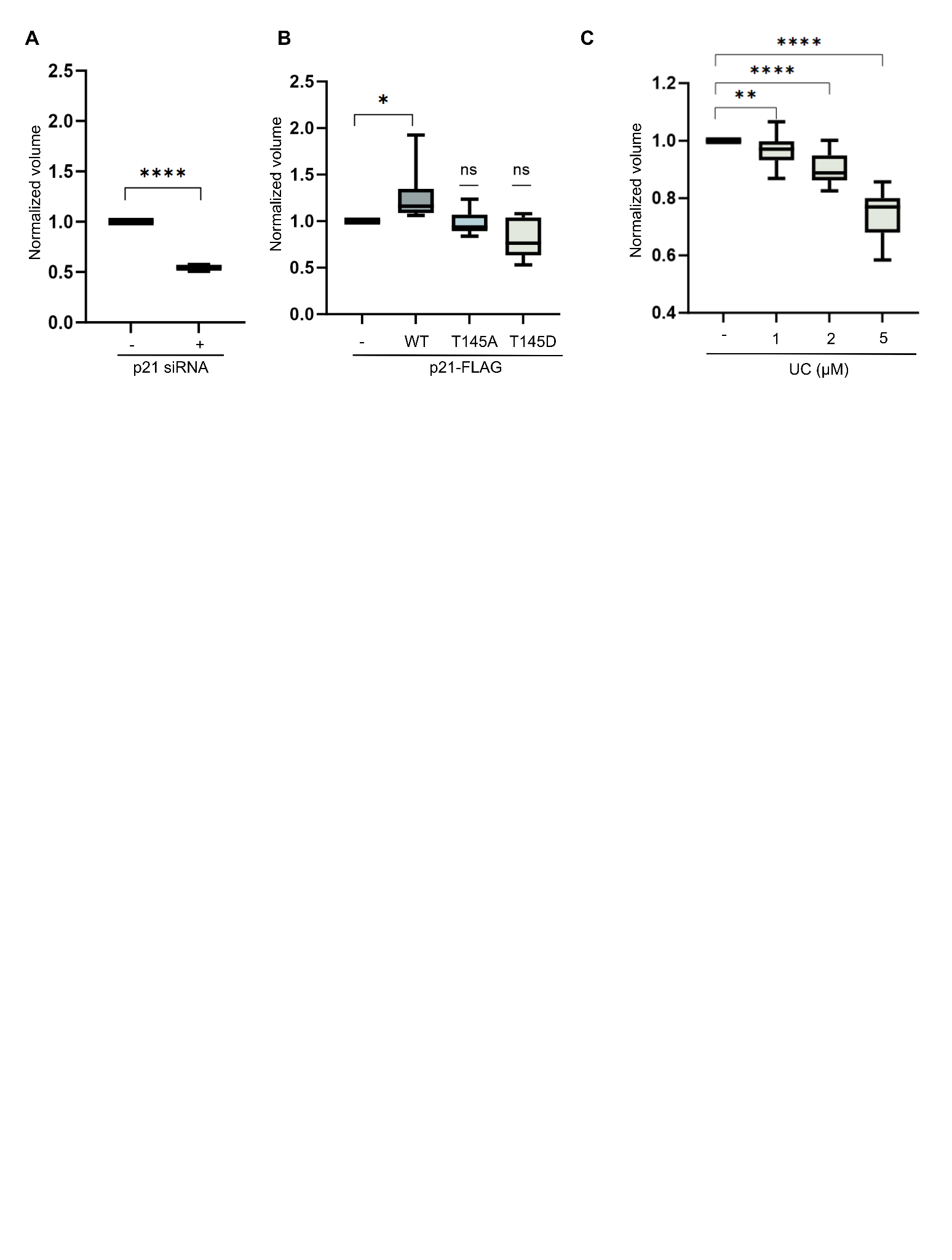
**

**Supplementary Fig. 3: Spheroid growth analysis of BT474 cells**

**A. Growth analysis of spheroids 14 days after their plating in a 96 well low adherent plate for CDKN1A/p21 knockdown cells compared with siRNA control cells normalized against day 1. At least 6 different wells were counted for each condition. B. Growth analysis of spheroids 14 days after their plating in a 96 well low adherent plate for cells overexpressing the p21-FLAG constructs compared with control (no transfected) cells normalized against day 1. At least 6 different wells were counted for each condition. C. Growth analysis of spheroids 8 days after culturing cells in low adherent 96 V bottom plates in DMSO (Control) or in the presence of UC2288 in various concentrations. Inhibitors were being added every day in the course of 8 days. The data are normalized against control (DMSO). For the experiment various separate wells were measured. Ns = non significant, **p*<0.05, ***p*<0.01, *****p*<0.0001.**

**
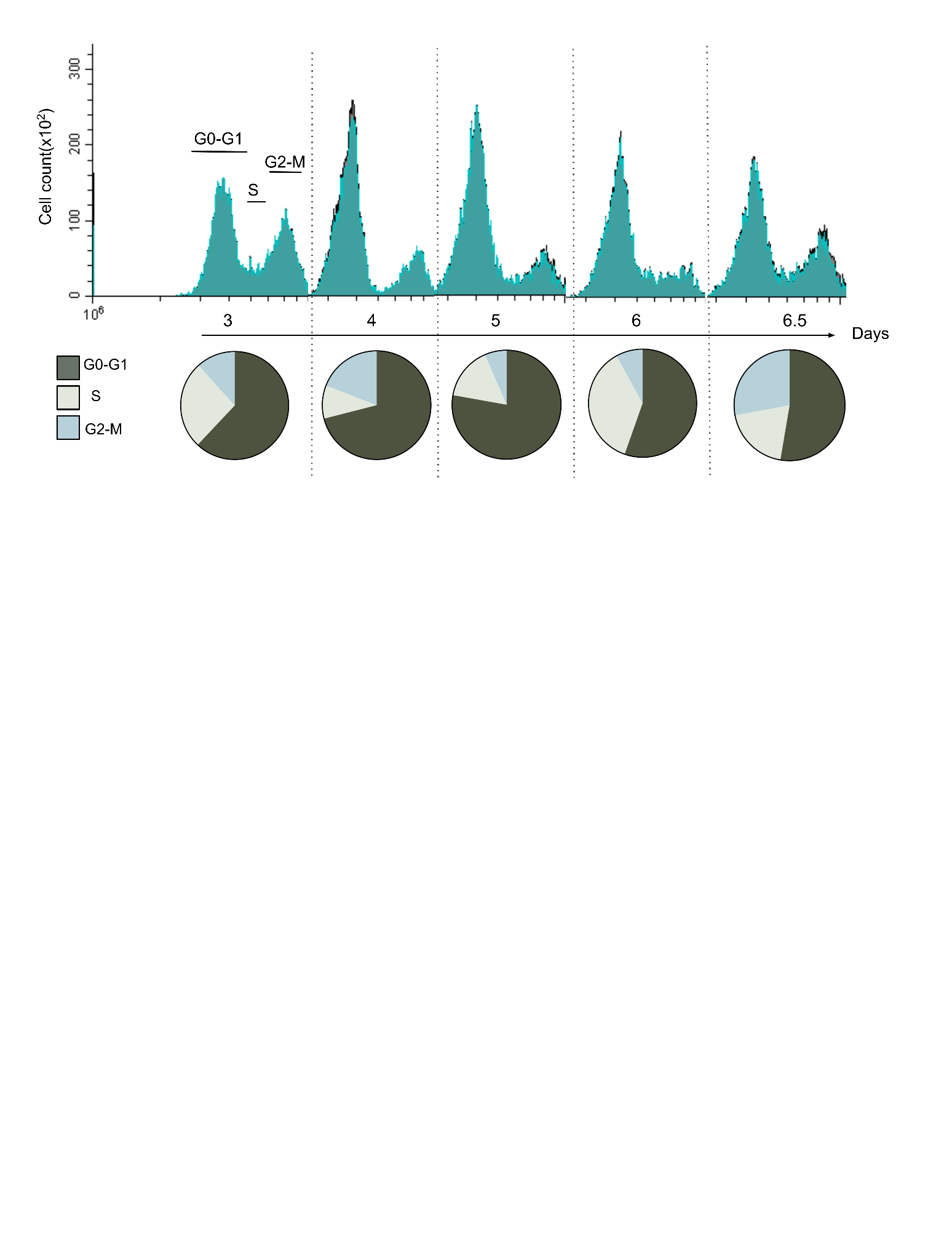
**

**Supplementary Fig. 4: Synchronization after serum deprivation of MCF7 cells.**

**FACS analysis with propidium iodide (PI) to determine the percentage of cells in each phase of the cell cycle on various days, and pie charts representing the percentage of each phase of the cell cycle through the different days of synchronization.**

**
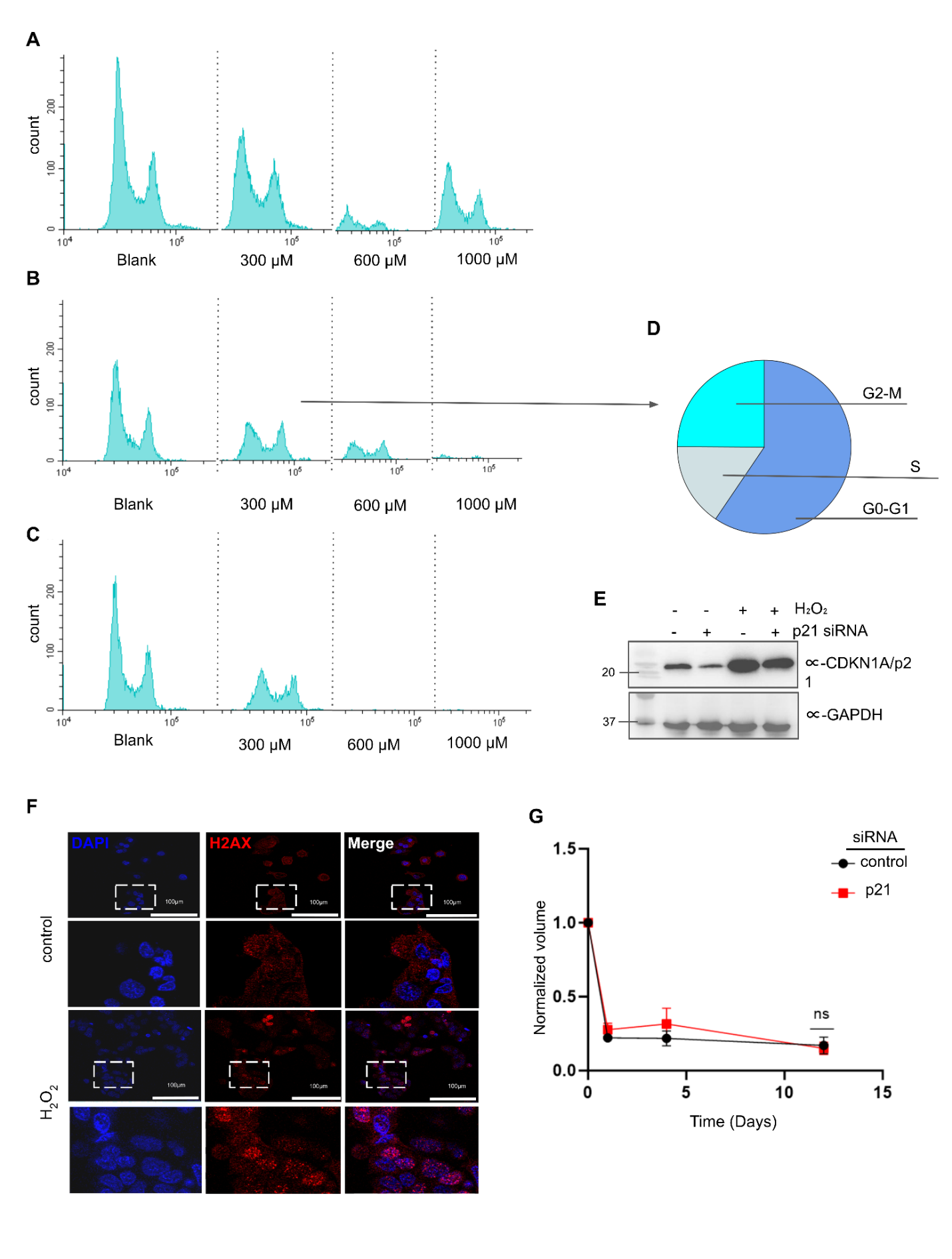
**

**Supplementary Fig. 5: Effect of H_2_O_2_ treatment on cell cycle and DNA damage**

**Histogram representing propidium iodide staining for MCF7 cells treated with H_2_O_2_ 300, 600, or 1000 μM for 3 hours (A), 6 hours (B), or 8 hours (C). D. Graphical representation of the percentage of cells that exist in each phase of the cell cycle after treatment with 300 μM of H_2_O_2_ for 6h (n=10000). G0-G1=47.1%, S=10% and G2-M=42.78%. The percentage is normalized by dividing the total number of cells in all the phases (total number of alive cells) with the number in each specific phase (n=1). E. Western blot analysis of the protein levels of CDKN1A/p21 24 hrs after treatment with 300μM H_2_O_2_ for 3 hrs in p21 KD or control cells. GAPDH was used as a loading control. F. Immunofluorescence analysis for γ-H2AX levels 6 hours after treatment with 300 μM H_2_O_2._ Scale bar = 100 μm; magnification = 63x. For both control and treated cells, an enlarged zoom is shown below, and the area enlarged is indicated using the dashed box. G. Growth rate of 12 days of CDKN1A/p21 knockdown cells or siRNA control cells growing in 2D monolayers treated with 300 μM of H_2_O_2_ for 6h. Ns, no significance.**


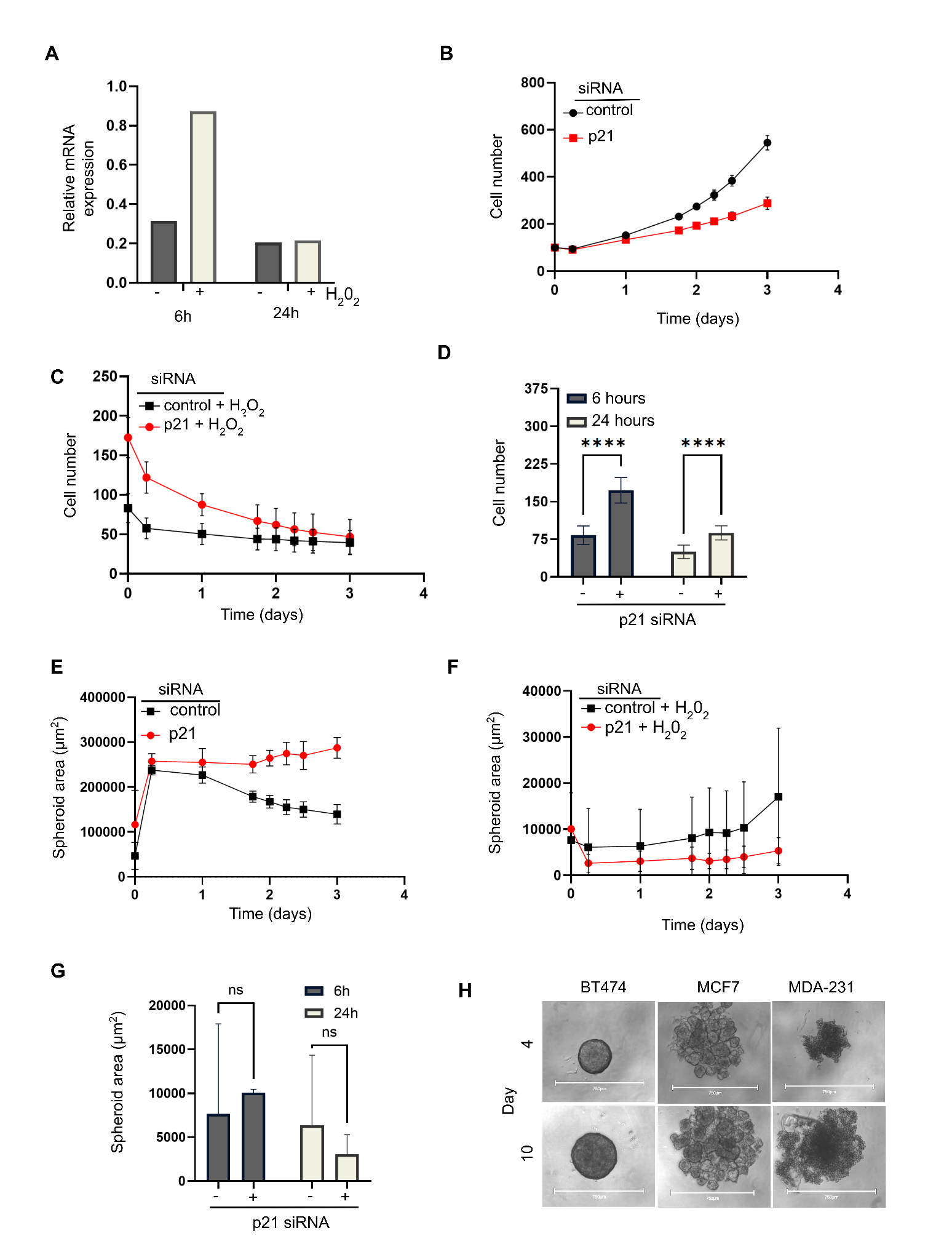


**Supplementary Fig. 6: Effect of CDKN1A/p21 KD on MDA-MB-231 cell growth and spheroid formation under oxidative stress. A. Relative mRNA expression of CDKN1A/p21 6 hrs after treatment with 300 µM H₂O₂ or 24 hrs after. B, C. Growth curves of CDKN1A/p21 KD compared with siRNA control cells under basal conditions (B) or after 6 hrs treatment with 300 µM H₂O₂ (C). D. Quantification of cell number 6 and 24 hrs after treatment with 300 µM H₂O₂. E, F. Growth of spheroids originated from CDKN1A/p21 KD cells or cells transfected with siRNA control under basal conditions (E) or after 6 hrs treatment with 300 µM H₂O₂ (F). G. Quantification of spheroid area 6 and 24 hrs after treatment with 300 µM H₂O₂. H. Representative brightfield images of spheroids formed by BT474, MCF7, and MDA-MB-231 cells transfected with control or CDKN1A/p21 siRNA and cultured for 4 or 10 days. Scale bar=750 µm; magnification=10x. ns: non-significant, *****p*<0.0001.**

**Supplementary Fig. 7**

**
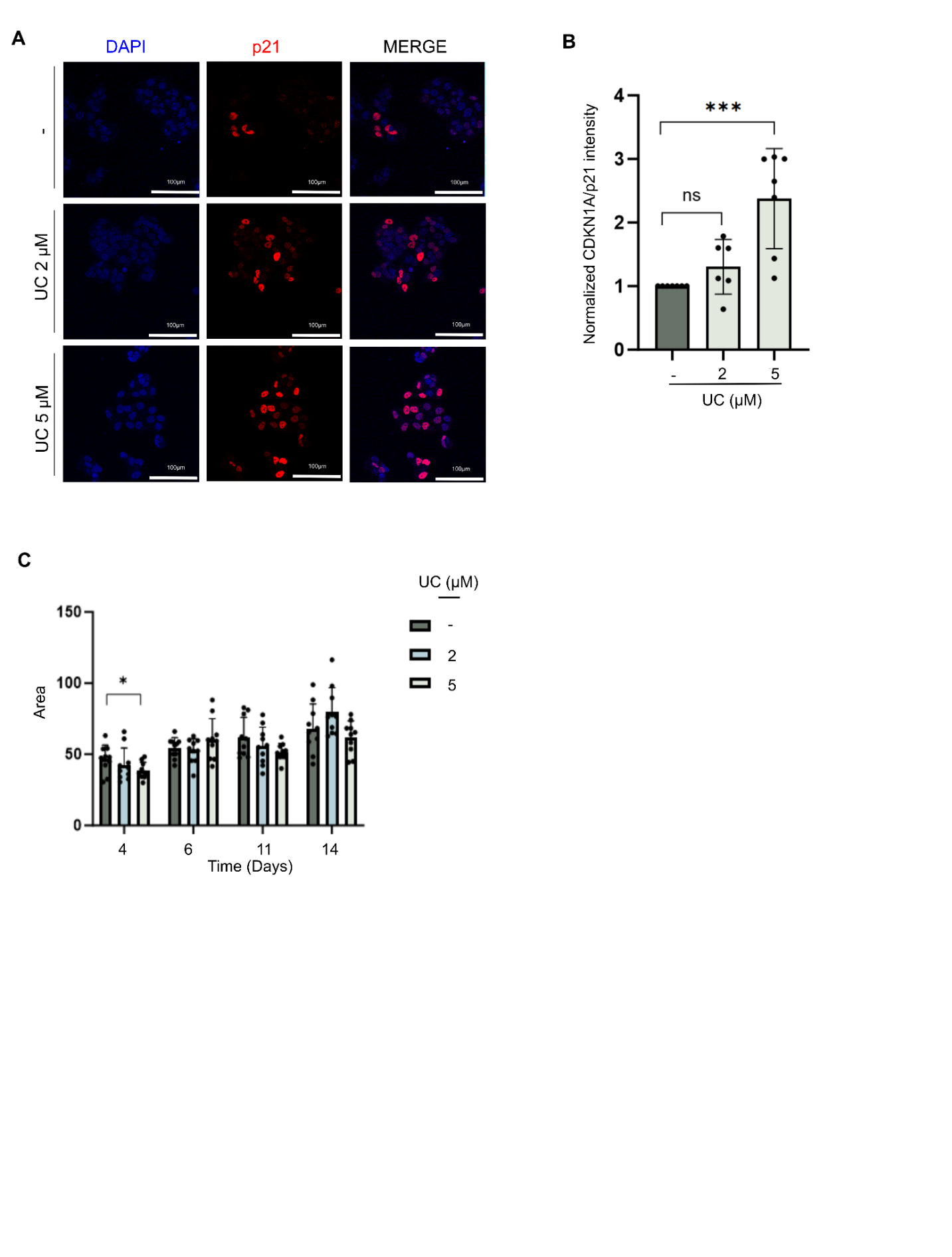
**

**Supplementary Fig. 7: Effect of UC2288 treatment on the growth rate of spheroids**

**A, B. Immunofluorescence analysis (A) and quantification (B) of the protein levels of CDKN1A/p21 after treatment with UC2288 (2 and 5 μM) or vehicle control (DMSO; indicated as ‘–’)). Scale bar = 100 µm. Magnification: 63x. C. Spheroid size measured in pixels² at days 4, 6, 11, and 14 for cells treated with UC2288 (2 and 5 μM). ns: non-significant, **p*<0.05, ****p*<0.001.**

**Supplementary Fig. 8**

**
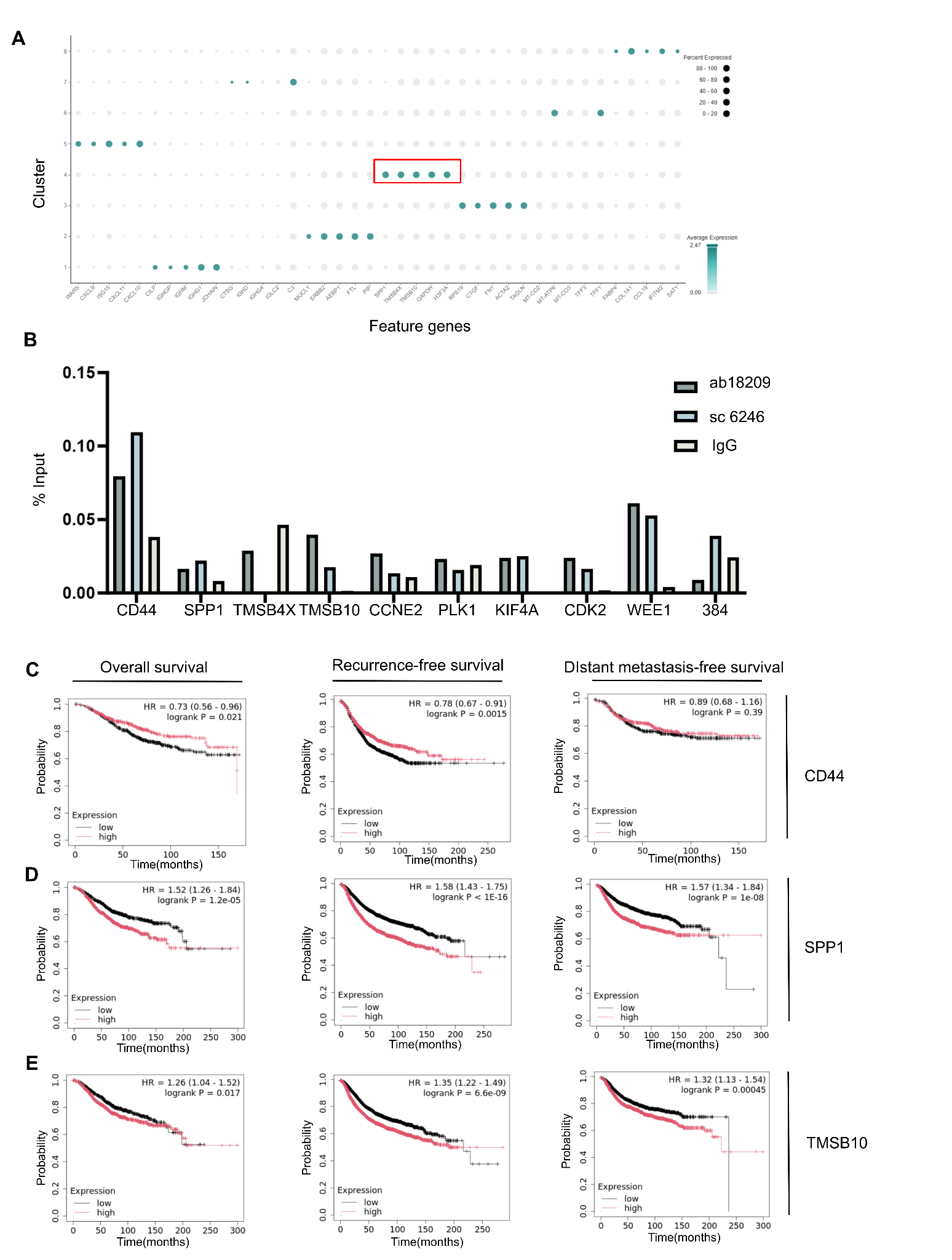
**

**Supplementary Fig.8. Spatial Transcriptomic Enrichment of CD44-Associated Genes, CDKN1A/p21 Promoter Binding, and Their Prognostic Significance in Breast Cancer.**

**A. Dot plot from Crost online tool for visualization of spatial transcriptomics data (GSE203612) showing the distribution of the most highly enriched genes across the different clusters. Cluster 4 enriched genes CD44, SPP1, TMSB4X, H3F3A, and TMSB10 are highlighted in a red box. B. Chromatin immunoprecipitation quantitative PCR (ChIP-qPCR) analysis of the DNA binding activity of CDKN1A/p21 in gene promoters using two different antibodies against CDKN1A/p21 (ab18209 and SC 6246). Enrichment of CD44, SPP1, TMSB4X, TMSB10, CCNE2, PLK1, KIF4A, CDK2, WEE1 promoters, and the randomly selected genomic region 384 compared with the input of the chromatin immunoprecipitation. IgG was used as a negative control. Kaplan-Meier curves of overall survival (OS), recurrence-free survival (RFS), and distant metastasis-free survival (DMFS) of breast cancer patients with high (red) or low (black) expression of CD44 (C), SPP1 (D), and TMSB10 (E). CD44 (OS *p*=2.1×10^-02^, RFS *p*=1.5×10^-03^, DMFS *p*=3.9×10^-01^), SPP1 (OS *p*=1.2×10^-05^, RFS *p*<1×10^-16^, DMFS *p*=1×10^-08^), TMSB10 (OS *p*=1.7×10**^-02^, RFS ***p*=6.6×10^-09^, DMFS*p*=4.5×10^-04^).**

**Supplementary Table 1: qPCR and ChIP-qPCR primers**

| **Name** | **Application** | **Forward (5’ to 3’)** | **Reverse (5’ to 3’)** |
| --- | --- | --- | --- |
| CDKN1A | qPCR | TTCTACCACTCCAAACGCCG | GGCAGAAGATGTAGAGCGGG |
| p53 | qPCR | TTGCGTGTGGAGTATTTGGAT | TGTAGTGGATGGTGGTACCAGTCAGA |
| cd44 | qPCR | ACTGAGACACGCAACCAAGAGGCA | CGTGAGGCTGCAGCTGTCCC |
| wee1 | qPCR | TACGGTGAAAGCTTGGGGAC | TGGGGACTATCACCACTTGC |
| PLK1 | qPCR | GCCCATGTGGGTTGAATGTG | CTCGTCGATGTAGGTCACGG |
| KIF4A | qPCR | TGGAAACAAGCAGTGTGGGT | CAGCCCTGATCCTACCTTGC |
| CDK2 | qPCR | CGTCAACGTGGGTCTTGGTA | ATCAGTGTGCCAACAGGGAG |
| CCNE2 | qPCR | GCCCAGTTCTGCATTACCCT | AGAAGAGAATGTCAAGACGAAGGT |
| PCNA | qPCR | ACCATCTTCCTGTCTTTCAGTTGT | CATCCTCGATCTTGGGAGCC |
| CCNE2 | ChIP-qPCR | TGATACATACCTGGGTGGGCG | TAAACACCACTCAGCCACGC |
| CDK2 | ChIP-qPCR | GGGTGCTCATTTGCATAGCC | GCAGGATGGGAAAGCAATGTC |
| KIF4A | ChIP-qPCR | CCCCACATTCTGCCTGAGAC | CCTCTTTTCGACCCTGCGGA |
| PLK1 | ChIP-qPCR | AGTGAACCGCAGGAGCTTTC | CAGCCGGGGAAAACCTGATT |
| WEE1 | ChIP-qPCR | TTGCGTTTGAGTTTGCCGC | GCCTCCGAGCCCCATTATTT |
| CD44 | ChIP-qPCR | AATTTGGCAGGGCGGGCACA | TTCCTCCAGCCCCACCACCC |
| SPP1 | ChIP-qPCR | GCTGAATGCCCATCCCGTAA | TCCCCCTCTGGTTTTGTGGT |
| TMBS4X | ChIP-qPCR | CACTGGTCCCCAAGAGCAAT | TGCATGCACCCTAACCACAA |
| TMSB10 | ChIP-qPCR | CACCCACGGCTACACATCAT | CCCCGCAGAAAGGATATGGG |

**Supplementary Table 2: Cancer Genome Atlas (TCGA) Abbreviations**

| Abbreviation | Full Name |
| --- | --- |
| acc | Adrenocortical carcinoma |
| aml | Acute Myeloid Leukemia |
| blca | Bladder urothelial carcinoma |
| brca | Breast invasive carcinoma |
| CRC | Colorectal cancer |
| esca | Esophageal carcinoma |
| LIHC | Liver cancer |
| luad | Lung adenocarcinoma |
| ov | Ovarian serous cystadenocarcinoma |
| paca | Pancreatic cancer |
| prad | Prostate adenocarcinoma |
| read | Rectum adenocarcinoma |
| raca¹ | Renal cancer Clear Cell |
| reca² | Renal cancer Chromophobe |
| reca³ | Renal cancer Papillary |
| skcm | Skin cutaneous melanoma |
| stad | Stomach adenocarcinoma |
| tgct | Testicular germ cell tumor |
| thca | Thyroid carcinoma |
| ucs | Uterine carcinosarcoma |
| ucec | Uterine corpus endometrial carcinoma |
